# FEDCODE: A Framework and Experimental Study of Model-Update Compression for Communication-Efficient Federated Learning

**DOI:** 10.3390/s26196297

**Published:** 2026-10-05

**Authors:** Emanuel Guberović, Igor Čavrak

**Affiliations:** Faculty of Electrical Engineering and Computing, University of Zagreb, 10000 Zagreb, Croatia; emanuel.guberovic@fer.unizg.hr

**Keywords:** federated learning, communication efficiency, model-update compression, adaptive quantization, entropy coding, edge computing

## Abstract

Federated learning trains shared models without centralizing local data, but repeated model exchange can become a bottleneck in bandwidth-, energy-, and latency-constrained edge systems. This paper presents FEDCODE, a communication-aware federated learning simulation framework for reproducible evaluation of update representations and compression methods with explicit accounting of communication volume and runtime under controlled training configurations. Experiments on MNIST, CIFAR-10, and LEAF-based federated datasets show that update representation, quantization scale, and coder choice strongly affect payload size, while lightweight integer and entropy coding can substantially reduce communication. Comparisons under a matched protocol with established FL compression methods show favorable communication–quality trade-offs for the evaluated FEDCODE delta-coding configurations. Adaptive coder-parameter tuning provides automatic parameter selection but does not consistently outperform strong fixed configurations. We further propose Coder-Aware Adaptive Delta Scaling (CAADS), which combines update statistics, drift in update magnitude, and coder-specific code-length estimation to adapt the quantization scale during training. CAADS adjusts the communication–quality trade-off: conservative settings preserve model performance, whereas aggressive scaling further reduces payload at the cost of larger quality degradation. Additional experiments on edge devices show that end-to-end compression benefits depend on device capability and network bandwidth. Overall, effective federated learning compression requires joint consideration of payload, model quality, codec cost, and network conditions.

## 1. Introduction

Federated learning (FL) [1] enables distributed devices to collaboratively train a shared model without transferring local training data to a central server. This property makes FL attractive for privacy-sensitive edge intelligence and mobile edge networks, where data is generated by end devices such as smartphones, embedded sensors, vehicles, and IoT nodes [2]. However, FL does not eliminate the need for communication: model parameters or model updates must still be exchanged between participating clients and the aggregation server during repeated training rounds.

This repeated exchange can become a practical bottleneck. When models are large, client populations are large, or many communication rounds are required, the transmitted payload can account for a substantial share of the overall training cost [1,3,4]. The problem is especially important in edge environments, where wireless bandwidth, energy availability, connectivity, and computational resources may be limited [2,5,6,7,8]. In addition, data distributions among clients often exhibit non-IID properties, affecting convergence and the communication needed to reach a desired model quality [9]. Communication-efficient FL therefore requires methods that reduce communication volume while preserving predictive performance and keeping additional system overhead acceptable [10].

Existing approaches address this challenge by reducing synchronization frequency, selecting or scheduling clients, transmitting fewer update values, or representing them with fewer bits [11,12]. Update compression focuses specifically on the transmitted model representation and can complement higher-level communication-control strategies. Lossy operations such as quantization reduce the amount of transmitted data but introduce a trade-off between communication rate and approximation distortion. Subsequent lossless coding stages can further reduce payload size, but they also introduce representation and processing overhead [13,14,15]. Communication efficiency should therefore be judged not only by compression ratio but also by computational overhead and the resulting model quality.

A further challenge is the limited comparability of results across compression studies. FL performance depends on the dataset and client partitioning [16,17], as well as on model architecture, aggregation behavior, client availability, system heterogeneity, and evaluation protocol [18]. For compression experiments, the measured communication cost additionally depends on the chosen update representation [3,19], quantization settings [15], payload encoding, metadata, and consistent accounting of both uplink and downlink communication [19,20]. Consequently, communication gains reported under different experimental configurations are difficult to compare directly. A controlled evaluation environment is therefore needed in which the learning process remains fixed while the transmitted representation and compression pipeline are varied systematically.

FEDCODE is a modular Python- and PyTorch-based framework [21] designed for studying communication-efficient model-update compression under controlled experimental conditions. The framework supports systematic variation of update representation, quantization settings, payload coders, and adaptive compression mechanisms while keeping the FL training and evaluation protocol fixed. It records mean per-client uplink payload, server-object downlink payload, coder metadata, and encoding and decoding times together with model-quality metrics. This enables joint analysis of communication reduction, computational cost, and model quality.

An initial version of FEDCODE was presented in our conference paper [22], which introduced the core framework and provided a preliminary single-seed evaluation of static compression methods and adaptive entropy-coding variants on MNIST and CIFAR-10. The present journal article substantially extends that work by introducing the Coder-Aware Adaptive Delta Scaling (CAADS) method, a broader multi-seed experimental methodology, more detailed communication and runtime accounting, and evaluations on additional update representations, quantization settings, federated Non-IID datasets, larger client populations, competitive FL-compression baselines, and edge-device hardware. Table 1 summarizes the main differences between the conference and journal versions.

The conference paper presented the initial framework demonstration. The present journal article introduces a new adaptive compression mechanism and provides substantially broader empirical validation covering parameter sensitivity, statistical heterogeneity, client-population scaling, edge-device execution, competitive baselines, and statistical robustness.

The key contributions of this work are as follows:

- An extended FEDCODE framework that supports controlled experiments on simulated and natively federated datasets, complemented by edge-device replay evaluation on Raspberry Pi hardware under varying effective network bandwidths.
- A comparative evaluation of quantization, static and adaptive integer and entropy coders, and matched-protocol FL-compression baselines (FedPAQ, FedBAT, and SparsyFed) covering quantization, learnable binarization, and sparsification.
- The Coder-Aware Adaptive Delta Scaling (CAADS) method, which adaptively selects the quantization scale for delta updates using update statistics and coder-specific code-length estimates.

The remainder of this paper is organized as follows. Section 2 reviews communication-efficient FL, update compression, adaptive compression, and reproducible evaluation. Section 3, Section 4, Section 5 and Section 6 present the system model, the FEDCODE framework, the compression pipeline, and the evaluated compression methods. Section 7 describes the experimental methodology, datasets, configurations, and evaluation metrics. The subsequent sections report and discuss the experimental results and conclude with directions for future work.

## 2. Related Work

Communication efficiency is one of the main challenges in federated learning, especially in edge and Internet of Things (IoT) environments where participating clients may face limitations in bandwidth, energy, latency, connectivity, and computational resources [2,5,23]. Existing research has addressed this challenge through several complementary approaches. Communication reduction and update compression involve strategies reduction and update compression involve strategies that modify the communication process through synchronization reduction, client selection, resource-aware coordination, and hierarchical aggregation, as well as methods that reduce the transmitted representation through quantization, sparsification, or sketching [2,3,5,24]. Payload coding of quantized model updates further reduces the transmitted representation by applying lossless coding to discrete update streams. Adaptive compression strategies adjust compression parameters during training in response to model, update, or system conditions [25,26]. Reproducible evaluation of communication-efficient FL provides controlled datasets, client partitions, and evaluation protocols for comparing federated learning methods [16,18]. This section reviews these directions from the perspective of the transmitted model-update representation, with emphasis on how exchanged updates are represented, compressed, coded, and evaluated.

### 2.1. Communication Reduction and Update Compression in Federated Learning

Communication-efficient federated learning methods can be broadly categorized into two complementary approaches that alter either the communication process or the transmitted representation.

Communication process-based approaches reduce communication by modifying its frequency, participants, topology, or routing. A decrease in synchronization frequency reduces the frequency of model information exchanges [1,24], while client-selection and scheduling methods determine which devices participate in each communication round [27,28,29]. For FL in highly resource-sensitive domains, methods that jointly optimize communication efficiency and on-device resource usage are proposed. To reduce communication costs in FL for wireless IoT networks, Chen et al. [30] propose a client scheduling policy that reuses stale local model parameters, along with an additional power and bandwidth allocation method.

Ribero et al. propose FLICS-OPT [31] for large-scale FL over heterogeneous clients with time-varying availability and additional client participation constraints driven by system objectives (energy efficiency, etc.). FLICS-OPT dynamically learns a client selection policy that adapts to client availability patterns and communication constraints, minimizing the negative effects of biased client sampling and data heterogeneity. Fine-grained Energy Planning Client Selection (FEPCS) [32] implements an energy-efficient client selection strategy in mobile edge Federated Learning (FL) environments. At the client level, FEPCS manages local training workloads by considering both residual energy and estimated transmission energy. At the system level, it utilizes a progressive participation rate and an energy budget deviation metric. These factors, combined with the clients’ data contributions, help to form an effective client selection scheme.

Topology-aware and hierarchical approaches modify the location and method of aggregation in federated learning. Hierarchical Federated Learning (HFL) introduces intermediate aggregation nodes between clients and the global server, enabling model updates to be aggregated closer to their source. This architecture can reduce communication with the cloud and take advantage of the organization of edge resources [33,34,35]. Additionally, communication-efficient HFL methods consider factors such as client-aggregator associations, aggregation frequency, heterogeneous resources, and client data distributions to improve communication organization [36,37]. The HierFLMC method [38] incorporates model compression techniques into a hierarchical framework, which improves communication efficiency between edge devices and cloud servers. Additionally, it introduces a preliminary soft clustering model update compression algorithm (SCMUC) that utilizes K-means for initial clustering, thereby reducing computational complexity on edge devices. Recent orchestration approaches explicitly treat communication costs as a runtime constraint, adapting the HFL configuration to respond to changes like client churn and infrastructure conditions [39]. Trindade et al. [40] propose two energy-aware client-selection methods for hierarchical federated learning (HFL): hierarchical energy-aware participant selection (HEPS-ML), which is a supervised approach based on energy, resources, performance, and data features; and hierarchical energy-aware participant selection using the sine cosine algorithm (HEPS-SCA), which balances accuracy, energy, and latency.

Update-compression methods reduce the amount of model representation transmitted during each communication event by employing techniques such as structured updates, quantization, sparsification, or sketching [3,24]. These methods primarily decrease communication by altering the information content, precision, or dimensionality of the updates being sent. Quantization modifies numerical precision, sparsification changes the set of transmitted coordinates, and sketching provides an approximate summary. Early work by Konečný et al. [3] proposed various strategies for minimizing uplink communication in FL, including structured and sketched representations, quantization, random rotations, and subsampling.

Structured updates allow edge devices to limit model updates to a smaller, parameterized subspace instead of transferring uncompressed models. An example of a structure-based algorithm is FedSCR [41], which reduces the number of parameters transmitted over the network while maintaining model accuracy. FedSCR aggregates parameter updates across channels and filters, identifies and removes redundant updates by comparing the aggregated values against a threshold, all while retaining the complete model. Additionally, the adaptive version of FedSCR dynamically adjusts the threshold to accommodate weight divergence in non-IID (Independent and Identically Distributed) data distributions, thereby enhancing the model’s robustness.

The Model Aggregation with Layer Divergence Feedback mechanism (FedLDF) [42] calculates the divergence between the local model and the global model from the previous training round. By utilizing layer-divergence feedback, this mechanism selects the distinct model layers that each client uploads to the central server, significantly reducing the data transferred in each training round. The FedNet2Net scheme [43] gradually increases model complexity. The server first trains a small model until its performance saturates, then it introduces increasingly complex models until the desired performance is achieved through function-preserving transformations. PPE-FL [44], a scheme designed for Internet-of-Unmanned Agents (IUA) in Federated Learning scenarios, replaces conventional gradient uploads with lightweight ranking-based votes. Moreover, PPE-FL supports robust aggregation under uncertain connectivity and agent dropout by enabling mask reconstruction through interactions among a subset of agents.

Quantization is one of the most widely used techniques in communication-efficient federated learning (FL) because it significantly reduces the number of bits required to represent each transmitted update value. Methods that combine Federated Averaging [1] with sparsification and/or quantization of model updates to a limited number of bits have successfully lowered communication costs with minimal impact on training accuracy [45]. Notable examples of these methods include QSGD-style quantization [46], HighVQ [47] and FedPAQ [24], all of which decrease update precision before transmission.

FEDQCS [48] sequentially performs block sparsification, dimensionality reduction, and quantization of local gradient vectors before they are transmitted to the parameter server. On the server side, parameter reconstruction is achieved by computing approximate Minimum Mean Square Error (MMSE) estimates of the local gradients.

FedVQCS [49] compresses local model updates from each client by first applying dimensionality reduction, followed by vector quantization. The parameter server then reconstructs the global model update using a sparse signal recovery algorithm applied to the aggregated compressed local model updates.

Recent approaches have integrated compression directly into the training process. For instance, FedBAT [50] employs binarization-aware local training to learn binary model updates during optimization, which helps reduce the approximation error associated with post-training binarization. Similarly, Chen et al. [51] introduced a nonuniform quantization scheme (NQFL) that effectively minimizes communication overhead in federated learning (FL) systems. This is achieved through a novel data preprocessing method combined with the Lloyd-Max algorithm. Additionally, Lyu et al. [52] propose stochastic quantization as an effective technique for secure and efficient federated learning. This method offers advantages in communication efficiency, data protection, and reduced signaling and computation overhead.

Federated Trained Ternary Quantization (FTTQ) [53] is a compression method derived from the Ternary Quantization (TTQ) algorithm, specifically adjusted for federated learning. It aims to reduce inference energy consumption on client devices by optimizing client-side quantized networks using a self-learning quantization factor. Additionally, the authors introduced a ternary federated averaging protocol (T-FedAvg) to minimize upstream and downstream communication in federated learning systems.

FedMQ+ [54] is a framework for heterogeneous federated learning that features enhanced dequantization. This framework accurately reconstructs full-precision weights from low-precision weights collected by the aggregator, employing a dequantization strategy based on normal functions. Experimental results indicate that FedMQ+ significantly improves convergence speed while maintaining comparable model accuracy and high communication efficiency.

Zheng et al. [55] conducted a separate analysis of uplink and downlink model transmissions and analyzed FedAVG convergence under non-IID dataset distributions, partial client participation, and finite-precision quantization. They found that efficient model transmission requires increasing the quantization level at a logarithmic rate, whereas transmitting weight differentials can use constant quantization levels.

Sparsification selectively transmits specific coordinates, such as thresholded or Top-*k* entries, while omitting the remaining update values [56]. This approach not only significantly reduces communication volume but also enables local training of complex models on resource-constrained edge devices  [57].

Time-correlated sparsification (TCS) strategy [14] relies on the assumption that important model parameters change slowly over time, using this correlation across training iterations to reduce communication load. Sparse Ternary Compression (STC) [19] combines sparsification, ternarization, and residual accumulation, and it additionally applies lossless encoding by transmitting nonzero positions and signs, representing positions as distances, and using Golomb coding.

The Federated Dynamic Sparse Training (FedDST) framework [58] allows dynamic extraction of sparse sub-networks from the original complex network, significantly reducing both on-device computation and in-network communication. PruneFL [59] introduces a low-complexity adaptive pruning method designed for edge and mobile devices. This method identifies the optimal model size that can achieve comparable prediction accuracy to the original model while significantly reducing processing time. Additionally, the proposed approach can be combined with other compression techniques, such as quantization, to further minimize communication overhead.

Shah and Lau [60] utilize various sparsification techniques for both upstream and downstream model compression, effectively reducing communication volume while maintaining model accuracy. The Federated Adaptive Deep Sparse Representation (FedADSR) [61] method incorporates an importance-based threshold mechanism that dynamically adjusts the pruning ratio for each model layer. This strategy reduces the computational burden on client devices and enhances overall system scalability.

Sketching methods such as FetchSGD and FedSketch use compact randomized summaries to reduce communication while approximately preserving key information about aggregate updates [62,63]. More recently, SparsyFed [64] has emerged, incorporating techniques like sparse local training, weight re-parameterization, adaptive activation pruning, and Top-K update pruning. Its adaptive sparse masks make SparsyFed particularly well-suited for heterogeneous cross-device federated learning (FL).

FEDCODE maintains a fixed FL execution protocol and examines how the representation of transmitted updates impacts communication costs, processing overhead, and model quality. It treats payload coding as a distinct, lossless stage applied after the update has been quantized. This separation allows for a clear distinction between the approximation introduced by the update representation and quantization and the additional byte reduction achieved through the lossless encoding of the resulting integer symbols.

### 2.2. Payload Coding of Quantized Model Updates

Once model parameters or updates have been quantized, they can be represented as streams of signed integers. These streams can then be transformed into nonnegative symbols through signed-to-unsigned mappings and encoded using lossless integer or entropy coders. This view is particularly relevant for delta-update transmission, where clients transmit changes relative to the current global model.

Prior empirical studies have shown that FL client updates commonly exhibit sparse, zero-centered, and heavy-tailed structure that can be effectively exploited by compression methods [4]. Following scaling and rounding, the resulting discrete stream may therefore contain a high concentration of zeros and low-magnitude symbols. Such distributions are well-suited for coding methods where codeword lengths depend on the magnitude of the symbols or their empirical frequency.

Universal integer codes, such as Elias-γ and Elias-δ, provide self-delimiting representations for positive integers without requiring a learned distribution or transmitted codebook [65]. Parameterized integer codes, such as Golomb–Rice and Exponential-Golomb coding, introduce tunable parameters that can be adjusted to better match different integer-magnitude distributions [66,67,68,69]. Byte-oriented variable-length coding, such as LEB128 [70,71], provides a practical alternative with a simple implementation and favorable runtime properties. Huffman coding provides a distribution-sensitive entropy coder that assigns shorter codewords to more frequent symbols, but it requires codebook construction and metadata accounting [72].

The tunable parameters of Golomb–Rice, Exponential-Golomb, and related integer-code families also make these coders suitable for adaptive parameter selection. Adaptive Golomb–Rice variants have been used in practical lossless coding systems, including adaptive run-length/Golomb–Rice coding and JPEG-LS-style residual coding, where coding parameters are adjusted to match changing source statistics [73,74]. In FEDCODE, this principle is applied to adaptive coder-parameter selection: for a fixed quantized FL update stream, the coding parameter is selected according to the current payload distribution before encoding.

The benefit of reducing transmitted data must be considered together with the processing required to perform that reduction, particularly within resource-limited IoT and edge environments. Research on communication reduction across the IoT–edge–cloud continuum distinguishes compression, aggregation, and prediction as complementary mechanisms and emphasizes that their suitability depends on processing complexity, communication requirements, energy consumption, and data characteristics [75]. Communication overhead can also arise at different layers: communication technologies and application-layer protocols affect protocol-related overhead, whereas the compression mechanisms considered here reduce the amount of application data that must be transported. FEDCODE focuses on the latter and keeps the underlying transport mechanism outside the coding comparison.

The coders discussed in this work are traditional data compression methods and are not new federated learning (FL) algorithms themselves. Their significance stems from the perspective of viewing quantized FL updates as integer data streams. From this perspective, communication costs are influenced not only by the number of bits used for quantization but also by the distribution of the resulting integer symbols, the coding method employed, coder metadata, and the overhead associated with encoding and decoding.

Previous work on FL compression has utilized lossless coding in specific contexts, such as encoding sparse update indices or position distances, using methods like STC [19]. However, there has been less focus on systematically comparing various integer and entropy coders for quantized full- and delta-update streams. This includes evaluating communication reduction, codec runtime, metadata overhead, uplink/downlink costs, and model quality under the same FL protocol.

### 2.3. Adaptive Compression Strategies

Static compression methods use fixed parameters throughout the training process, including a constant quantization level, sparsity ratio, sketch size, or coding parameter. While this approach enhances reproducibility, it can be less effective since the distribution of federated learning (FL) updates, model states, client behaviors, and communication conditions may change across different rounds. Adaptive compression methods tackle this problem by adjusting compression parameters dynamically during training.

AdaQuantFL [25] adapts the number of quantization levels throughout the training process to enhance the balance between error and communication. This approach utilizes a schedule derived from convergence analysis regarding the number of bits communicated. DAdaQuant [26] builds upon this concept by adjusting quantization not only over time but also across clients, aiming to minimize communication from client to server without compromising model quality. AQUILA [76] integrates adaptive quantization with a device-selection strategy, leveraging update usefulness and model deviation criteria to enhance communication efficiency in heterogeneous federated learning (FL) environments. Other methods adjust compression based on fluctuating network conditions; for instance, AdapComFL [77] forecasts client bandwidth and modifies sketch-based model compression in accordance with predicted uplink capacity.

The FedDG framework [78] introduces the AdaSTC method, which adaptively adjusts the client compression rate based on model training errors, reducing transmission costs while improving convergence speed and accuracy. In addition, to mitigate the impact of low-resource clients and reduce communication overhead, the method uses dynamic client selection based on training time and model performance.

Chen et al. [51] propose an adaptive quantization approach that considers both the correlation of gradients among local clients and the correlation of gradients between communication rounds. The compression strategy is based on rate-distortion theory and the Kalman filter, allowing determination of an optimal gradient quantization strategy.

Yun et al. propose a FedSpar [79], communication-efficient FL framework with improved convergence under limited uplink capacity. The framework is based on a classic top-S sparsification approach with quantization and compression of uplink data. However, it minimizes compression (client) and reconstruction (aggregator) errors by linearly transforming update values to model them as Gaussian random variables, optimizing the quantizer design for value encoding, and employing an LMMSE-based reconstruction method on the aggregator side.

Adaptation can occur beyond just the compression mechanism itself. In hierarchical federated learning (FL), runtime orchestration can adjust client-aggregator associations or aggregation behavior as communication conditions and client availability change [39]. This highlights the broader distinction between adapting the communication process and modifying the representation of the transmitted payload.

Current adaptive methods primarily change various parameters such as quantization resolution, sparsity, sketch size, and other compression-related factors based on training progress, client characteristics, update information, or communication conditions. In the context of payload coding addressed here, adaptation can occur at two distinct stages: first, the parameters of the lossless coder can be selected for a stream that has already been quantized; second, the quantization scale itself can be adjusted before coding. FEDCODE evaluates the first case through adaptive selection of coder parameters and introduces the second as a methodological contribution of this work.

CAADS differs from prior adaptive quantization approaches in the criterion used to determine the transmitted numerical representation. Rather than adapting quantization resolution solely from training progress, client-specific conditions, or update statistics, CAADS combines an update-informed scale proposal with a downstream coder-specific objective. Quantization-induced zero fraction and update magnitude determine a scale region appropriate for the current client update, after which candidate scales are evaluated according to the estimated code length of the selected payload coder subject to a zero-fraction constraint. The scale is therefore adapted not only to the numerical characteristics of the current update but also to how the resulting discrete representation is expected to be encoded.

### 2.4. Reproducible Evaluation of Communication-Efficient FL

Reproducible federated learning (FL) experimentation requires controlled datasets, client partitions, model configurations, aggregation rules, and evaluation protocols. LEAF introduced datasets and evaluation settings that include realistic user-level partitions, along with statistical and systems metrics for federated environments [16]. Other frameworks like FedScale, FedML, and TensorFlow Federated offer broader support for FL simulation, benchmarking, and algorithm development [18,80,81]. PyTorch also provides the underlying tensor-computation and automatic-differentiation infrastructure that many experimental machine learning systems rely on [21].

These frameworks enhance repeatability and facilitate the evaluation of FL algorithms in realistic or standardized environments. However, they are not primarily designed for detailed analysis of the transmitted update representation. When conducting compression studies in edge and Internet of Things (IoT) settings, practical evaluation must consider not only the volume of transmitted data but also the overhead from representation, codec metadata, and encoding and decoding costs. Additionally, communication direction and frequency must be factored in. Therefore, achieving a favorable compression ratio alone may not be sufficient if the associated processing or representation overhead is considerable [75].

In particular, systematic comparison of quantization settings, integer payload coders, metadata overhead, encoding time, decoding time, and uplink/downlink payload accounting had not been fully supported as a single controlled compression-evaluation pipeline by the existing frameworks. This limitation motivated the creation of FEDCODE as an evaluation setting in which the learning configuration remains fixed while the transmitted update representation, quantization, payload coding, metadata, and runtime overhead can be examined systematically.

## 3. System Model and Problem Formulation

This work considers a federated learning system based on the FedAvg method [1], composed of a central aggregation server and a set of edge clients. Each client owns a local dataset that remains local and is not transferred to the server. Collaborative learning is therefore achieved through repeated communication rounds in which selected clients train locally and exchange model information with the server.

Let K={1,…,K} denote the set of available clients. In communication round *t*, the server holds the current global model parameters $w^{t}$. A subset of clients $S^{t}\subseteq K$ is selected to participate in the round. The server provides the current global model, or an encoded representation of it, to the selected clients. This communication direction is referred to as the *downlink*. Each selected client $k\in S^{t}$ initializes local training from $w^{t}$ and performs one or more local optimization steps on its private dataset $D_{k}$. After local training, client *k* obtains the local model parameters $w_{k}^{t+1}$.

The client may transmit either the full local model parameters or a difference update, also referred to as a delta update, relative to the received global model. In the full-update case, the transmitted object is

(1) $$u_{k}^{t}=w_{k}^{t+1}.$$

In the delta-update case, the transmitted object is

(2) $$u_{k}^{t}=\Delta w_{k}^{t}=w_{k}^{t+1}-w^{t}.$$

The client sends this object back to the server through the *uplink*. The server decodes the received client payloads, reconstructs the transmitted objects, and aggregates them according to the FedAvg aggregation rule to obtain the next global model.

**Figure 1:**
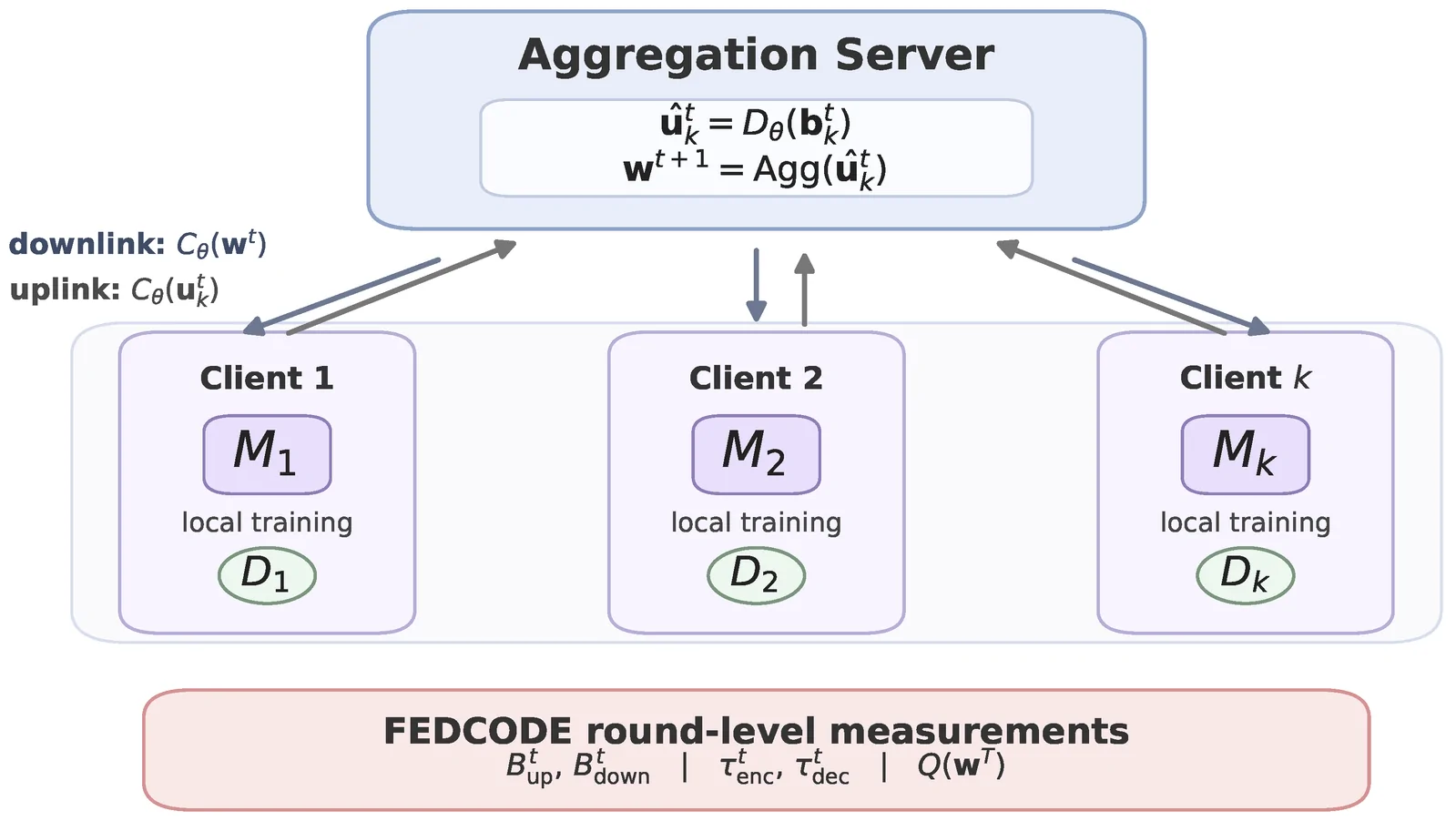
System model and FL communication flow considered in this work. The aggregation server distributes the current global model to selected clients via the downlink; clients perform local training on private datasets and return compressed client updates via the uplink. The considered measurements include mean per-client uplink payload, server-object downlink payload, encoding and decoding overhead, and final model quality.

Figure 1 illustrates the FL communication process considered in this work, including downlink transmission of the global model, local client training, uplink transmission of client updates, server aggregation, and the round-level communication and processing measurements.

Let $n_{k}=\left|D_{k}\right|$ denote the number of local training samples on client *k*. The aggregation weight of client *k* in round *t* is defined as

(3) $$\alpha_{k}^{t}=\frac{n_{k}}{\sum_{j\in S^{t}}n_{j}}.$$

In the full-update case, the server aggregates the reconstructed local model parameters directly:

(4) $$w^{t+1}=\sum_{k\in S^{t}}\alpha_{k}^{t}{\hat{w}}_{k}^{t+1}.$$

In the delta-update case, weighted federated averaging updates the global model as

(5) $$w^{t+1}=w^{t}+\sum_{k\in S^{t}}\alpha_{k}^{t}{\hat{\Delta w}}_{k}^{t},$$

where ${\hat{w}}_{k}^{t+1}$ denotes the reconstructed local model parameters of client *k*, and ${\hat{\Delta w}}_{k}^{t}$ denotes the reconstructed client delta update, both after compression and decompression.

When all selected clients have the same number of local training samples, the weights reduce to

(6) $$\alpha_{k}^{t}=\frac{1}{|S^{t}|},$$

and the weighted aggregation becomes simple averaging over the selected clients. The considered system uses the sample-weighted formulation when client sample counts are available and falls back to simple averaging when they are not.

Both uplink and downlink communication directions can be compressed. Uplink compression reduces the size of client-to-server transmissions. It directly addresses a major scalability bottleneck in federated learning, since many resource-constrained clients must transmit locally trained model updates over limited wireless connections. Downlink compression reduces the cost of distributing the global model, or a server-side update representation, from the server to selected clients. In practical edge- and sensor-oriented systems, both communication directions matter: uplink traffic is constrained by limited client transmission capacity, wireless resources, and energy use, while the downlink distribution of the global model contributes to round latency and scalability when many clients must receive updated parameters [2,5].

The training process is constrained by bandwidth, latency, energy use, and compression overhead. In synchronous FL, a communication round cannot complete until the selected clients receive the current model, perform local training, encode and transmit their updates, and the server decodes and aggregates the received information. Therefore, a compression method should be evaluated not only according to the resulting payload size but also according to the processing overhead required for encoding and decoding and its effect on predictive performance.

This work studies compression as a transformation applied to the transmitted model object. Let $C_{\theta }$ denote a compression method with configuration parameters θ, and let $D_{\theta }$ denote the corresponding decompression method. For a client-side transmitted object $u_{k}^{t}$, which may represent either full model parameters or a delta update, the transmitted payload is

(7) $$b_{k}^{t}=C_{\theta }\left(u_{k}^{t}\right),$$

where $b_{k}^{t}$ is the byte stream transmitted over the communication channel. The server reconstructs the transmitted object as

(8) $${\hat{u}}_{k}^{t}=D_{\theta }\left(b_{k}^{t}\right).$$

The reported uplink payload of round *t* is expressed as the mean encoded payload per participating client:

(9) $${\bar{B}}_{\mathrm{up}}^{t}=\frac{1}{|S^{t}|}\sum_{k\in S^{t}}\left|b_{k}^{t}\right|.$$

The corresponding total round uplink is $B_{\mathrm{up},\mathrm{total}}^{t}=\left|S^{t}\right|{\bar{B}}_{\mathrm{up}}^{t}$. For downlink accounting, the server-side object is compressed once per round. Depending on the downlink protocol, this object is either the updated global model parameters or an aggregated update representation. Let $b_{\mathrm{server}}^{t}$ denote the corresponding compressed server-side payload. The reported server-side downlink object size is

(10) $$B_{\mathrm{down},\mathrm{obj}}^{t}=\left|b_{\mathrm{server}}^{t}\right|.$$

This metric represents the size of the compressed object produced by the server and does not necessarily correspond to the total physical traffic generated by a particular network deployment. For example, if the server transmits the same object separately to each selected client using unicast communication, the corresponding network-level downlink traffic is

(11) $$B_{\mathrm{down},\mathrm{unicast}}^{t}=\left|S^{t}\right|\;B_{\mathrm{down},\mathrm{obj}}^{t}.$$

In contrast, broadcast or multicast mechanisms may distribute the same server-side object without generating a separate full transmission for every selected client. Reporting the encoded server-side object size separately therefore allows the compression method to be evaluated independently of a specific physical downlink delivery mechanism.

The goal of the considered compression process is to reduce the reported representation-level communication cost while preserving model quality and keeping compression overhead acceptable. This trade-off can be expressed through the following constrained optimization objective:

(12) $$\min_{\theta }\sum_{t=1}^{T}\left({\bar{B}}_{\mathrm{up}}^{t}+B_{\mathrm{down},\mathrm{obj}}^{t}\right),$$

subject to a predictive-performance constraint

(13) $$Q\left(w^{T}\right)\geq Q_{\mathrm{ref}}-\epsilon ,$$

and an execution-overhead constraint

(14) $$\tau_{\mathrm{enc}}^{t}+\tau_{\mathrm{dec}}^{t}\leq \tau_{\max},\;t=1,\ldots ,T,$$

where $Q(w^{T})$ denotes the final model quality metric, such as model accuracy, loss, or perplexity; $Q_{\mathrm{ref}}$ denotes the quality obtained by a reference uncompressed or baseline quantized run; ϵ defines the acceptable degradation relative to that reference; and $\tau_{\mathrm{enc}}^{t}$ and $\tau_{\mathrm{dec}}^{t}$ denote the encoding and decoding overhead in round *t*.

The formulation presented above describes the desired trade-off between communication quality and processing, rather than posing a direct optimization problem addressed in this work. Instead, it establishes the evaluation objective for comparing different compression configurations. The framework introduced in the following section provides the tools needed to apply and evaluate these configurations. It does so by measuring factors such as mean uplink payload size per client, downlink payload size per server object, compression rate, encoding and decoding time, and the final model quality. Throughout this process, the local training procedure, client sampling policy, aggregation rule, and model-dataset configuration remain fixed.

## 4. FEDCODE Framework

Building on the system model and evaluation objective defined in Section 3, the extended FEDCODE framework is used to configure and execute FL simulations, construct full or delta model updates, apply static and adaptive compression to transmitted objects, and collect communication, runtime, and learning metrics under controlled experimental configurations. The extended FEDCODE implementation is written in Python using PyTorch [21] and is designed for communication-aware FL evaluation. For each experiment, the dataset, model architecture, client selection policy, aggregation rule, local training configuration, update representation, and compression method are specified explicitly. This allows raw transmission, quantization-only transmission, static integer and entropy coding, and adaptive compression methods to be evaluated under the same FL training configuration. The framework records byte-level mean per-client uplink and server-object downlink communication costs, encoding and decoding times, compression rates, model-performance metrics, run configurations, and round-level results. Deterministic client sampling supports repeated experiments with the same participating clients, while checkpointing allows longer simulation runs to be resumed from an intermediate state.

**Figure 2:**
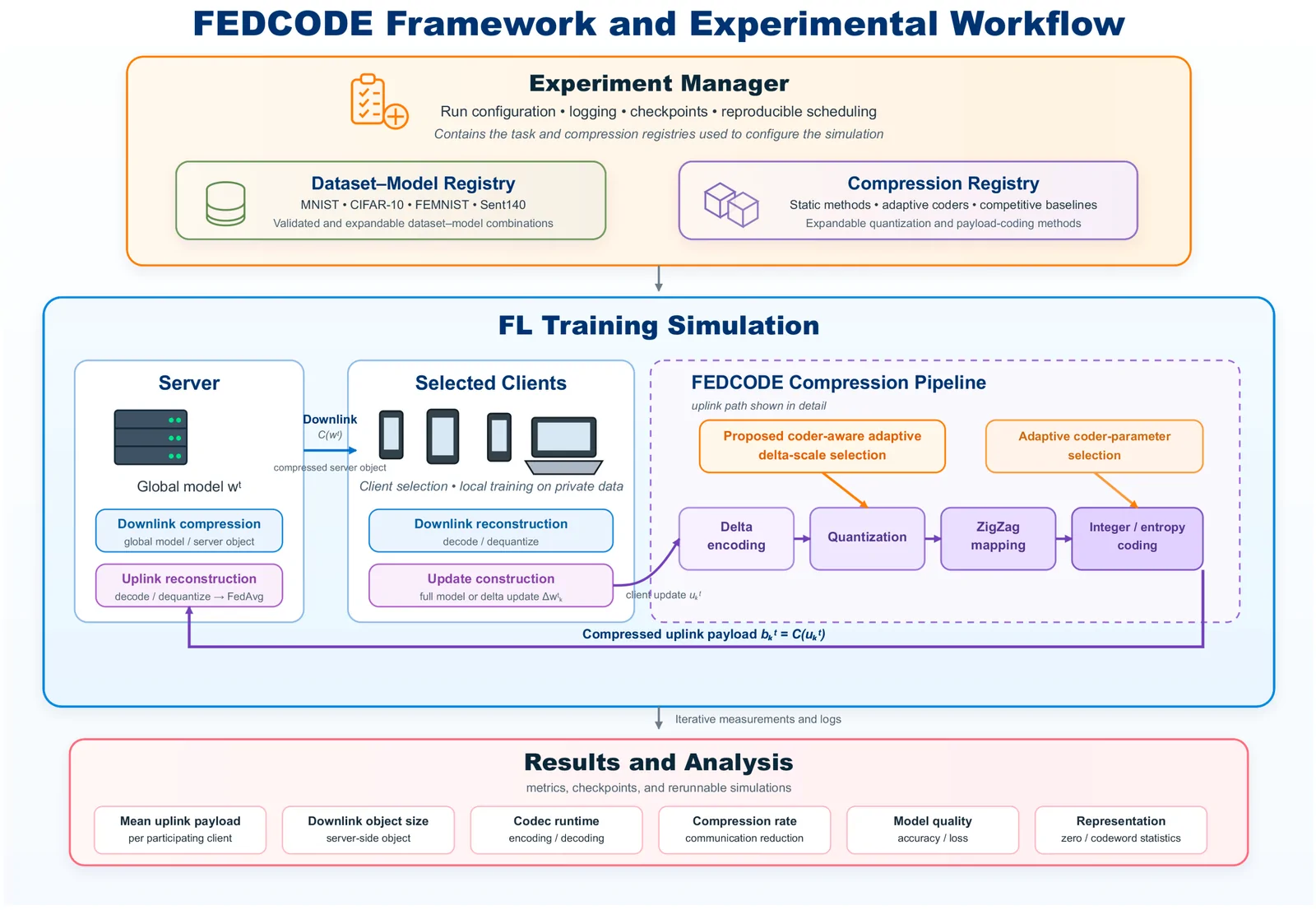
Architecture and experimental workflow of the FEDCODE framework. The Experiment Manager manages execution through run configuration, logging, and checkpointing. The FL Training Simulation integrates the federated learning core with the Dataset–Model and Compression registries, supporting local training, compressed model exchange, and aggregation. Results and Analysis collect communication, runtime, and model-quality measurements for subsequent evaluation.

As illustrated in Figure 2, FEDCODE is structured around three main functional components: the Experiment Manager, the FL Training Simulation, and Results and Analysis management. The FL Training Simulationis further divided into the Federated Learning Simulation Core, the Dataset–Model Registry, and the Compression Registry.

The Experiment Manager handles run configuration, logging, and checkpointing, and provides the configuration used to instantiate a simulation. Within the FL Training Simulation, the Dataset–Model Registry provides the dataset and model definitions associated with the experiment. The Federated Learning Simulation Core executes the communication rounds, including client selection and local training, compressed client–server model exchange, and server-side aggregation. The Compression Registry provides the compression methods applied to the model information exchanged during these rounds, including delta encoding, quantization, and integer or entropy coding with static or adaptive configurations. The resulting communication, runtime, and learning measurements are passed to Results and Analysis for evaluation.

During FL simulation, the Federated Learning Simulation Core obtains the configured learning task from the Dataset–Model Registry and processes transmitted model objects using methods from the Compression Registry. Depending on the experiment configuration, the transmitted object may consist of full model parameters or a delta update. The corresponding compression pipeline can combine quantization, signed-to-unsigned mapping, integer or entropy coding, and adaptive parameter selection. The detailed compression pipeline and evaluated methods are described in Section 5 and Section 6.

In the evaluated pipelines, quantization maps floating-point values to integer symbols, while the subsequent payload coder losslessly encodes the resulting integer stream. For delta transmission, the quantization scale determines the mapping between the floating-point update and its integer representation. FEDCODE additionally implements CAADS, in which the quantization-induced zero fraction and root-mean-square update magnitude are combined to obtain an update-informed scale proposal, which is subsequently refined using coder-specific code-length estimates. For parameterized integer coders, coding parameters can likewise be selected from the current integer stream according to their code-length functions. These mechanisms allow evaluation of fixed configurations, coder-parameter-adaptive configurations, and the proposed scale-adaptive method within the same FL training procedure.

The source code, experiment scripts, and run configurations used for the reported experiments are publicly available https://github.com/Guber/fed-code (accessed on 27 September 2026). The repository contains the resolved configurations for the reported experiments, including dataset–model selections, FL hyperparameters, compression settings, quantization parameters, random seeds, and analysis scripts.

In this study, we utilize FEDCODE to isolate the impact of different transmitted update representations and compression techniques while maintaining a controlled federated learning (FL) configuration. We compare compression configurations based on communication, processing, and model-quality measurements, as outlined in Section 3. The subsequent sections will detail the compression pipeline and the specific compression methods employed in these comparisons.

## 5. Compression Pipeline

**Figure 3:**
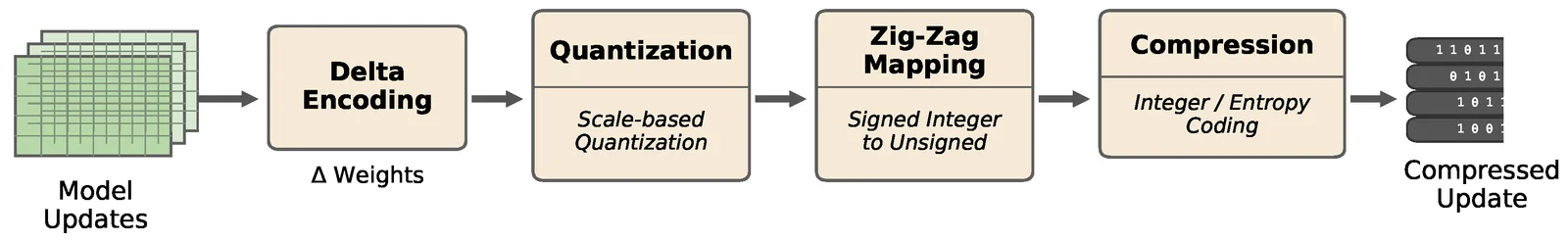
Overview of the FEDCODE compression pipeline. A transmitted model object is represented either as full model parameters or as a delta update. Floating-point values are quantized to signed integers, mapped to nonnegative symbols using ZigZag encoding, and encoded into a compressed byte stream. Decoding applies the corresponding operations in reverse.

The extended FEDCODE update-compression pipeline integrates delta encoding, quantization, signed-to-unsigned mapping, and either integer or entropy coding into the FL communication workflow. As summarized in Figure 3, it transforms a model object into a compressed byte stream through update construction, quantization, integer mapping, and payload coding. Individual methods are implemented by configuring specific pipeline stages, including the quantization scheme, payload coder, and adaptation strategy.

As a running example, consider three model parameters. Let the global model at round *t* be

$$w^{t}=\left[0.50,\;-0.20,\;0.10\right],$$

and let the locally trained model produced by client *k* be

$$w_{k}^{t+1}=\left[0.51,\;-0.22,\;0.10\right].$$

### 5.1. Update Representation and Delta Encoding

During each communication round, FEDCODE prepares the data that each selected client sends to the server. It supports two types of update representations: full-update mode and delta-update mode. In full-update mode, the client sends the complete model parameters that it has locally trained. In delta-update mode, the client transmits only the differences between its local model parameters and the global model parameters it received at the start of the round.

For client *k* in round *t*, the delta update is defined as

(15) $$\Delta w_{k}^{t}=w_{k}^{t+1}-w^{t}.$$

Delta encoding does not reduce the number of transmitted parameters; rather, it changes their distribution. Since local training usually adjusts the received global model incrementally, delta values often have smaller magnitudes than the full model parameters. This distributional shift can benefit subsequent quantization and integer coding.

For the running example,

$$\begin{array}{cc} \Delta w_{k}^{t} & =[0.51,\;-0.22,\;0.10]-[0.50,\;-0.20,\;0.10]\\ & =[0.01,\;-0.02,\;0.00]. \end{array}$$

### 5.2. Quantization and Scaling

Quantization reduces the numerical precision of transmitted values by mapping floating-point values to a finite set of integer representations. It is widely used in communication-efficient distributed learning to reduce the number of bits required to represent model parameters or updates [46].

In the considered pipeline, the floating-point update tensor is transformed into a fixed-width signed integer tensor. For a scalar update value *x*, fixed-scale quantization is defined as

(16) $$q=\mathrm{clip}\left(\mathrm{round}\left(\frac{x}{s}\right),q_{\min},q_{\max}\right),$$

where s>0 is the quantization scale and $\left[q_{\min},q_{\max}\right]$ is the representable integer range determined by the selected number of quantization bits. The scale determines the resolution of the quantized representation: a smaller scale preserves finer changes but can produce integer symbols with larger magnitudes, whereas a larger scale increases rounding and may map more values to zero.

With s=0.01, the running example becomes

$$\begin{array}{cc} \left[0.01,\;-0.02,\;0.00\right] & \to \left[\mathrm{round}\left(\frac{0.01}{0.01}\right),\mathrm{round}\left(\frac{-0.02}{0.01}\right),\mathrm{round}\left(\frac{0.00}{0.01}\right)\right]\\ & =[1,\;-2,\;0]. \end{array}$$

In this case, all three update values are exactly representable at the selected scale.

If a coarser scale is used, information may be lost before payload coding. For example, with s=0.05,

$$\begin{array}{cc} \left[0.01,\;-0.02,\;0.00\right] & \to \left[\mathrm{round}\left(\frac{0.01}{0.05}\right),\mathrm{round}\left(\frac{-0.02}{0.05}\right),\mathrm{round}\left(\frac{0.00}{0.05}\right)\right]\\ & =[0,\;0,\;0]. \end{array}$$

The resulting stream is potentially easier to encode since all symbols are set to zero; however, it eliminates the original parameter changes. This distinction matters in the pipeline being considered: quantization can introduce approximation errors, while the subsequent payload coder is lossless with respect to the quantized integer stream.

### 5.3. Signed-to-Unsigned Integer Mapping

After quantization, the update stream contains signed integers. Since the evaluated integer coders operate on nonnegative symbols, FEDCODE applies a ZigZag signed-to-unsigned mapping before payload coding. ZigZag encoding is used, for example, by Protocol Buffers for signed integers before variable-length integer encoding, mapping small positive and negative values to small nonnegative integers [82].

For a signed integer *q*, the mapping used in FEDCODE is

(17) $$z\left(q\right)=\left\{\begin{array}{cc} 2q, & q\geq 0,\\ -2q-1, & q<0. \end{array}\right.$$

The mapping interleaves nonnegative and negative values around zero:

0↦0, −1↦1, 1↦2, −2↦3, 2↦4.

Accordingly, the signed quantized vector from the running example,

[1, −2, 0],

is transformed into the unsigned symbol stream

[2, 3, 0].

This representation is useful for the evaluated integer coders because small positive and negative quantized values remain close to zero after mapping, allowing magnitude-sensitive coding schemes to represent them efficiently.

### 5.4. Payload Encoding and Reconstruction

The unsigned integer stream is passed to the selected payload coder, which produces the byte stream transmitted through the communication channel. Let $z_{k}^{t}$ denote the unsigned symbol stream obtained after quantization and ZigZag mapping. For a payload coder $E_{\phi }$ with coder configuration ϕ, the transmitted byte stream is

(18) $$b_{k}^{t}=E_{\phi }\left(z_{k}^{t}\right).$$

At the receiver, the corresponding decoder reconstructs the unsigned symbol stream. The inverse ZigZag mapping then recovers the signed quantized values, which are dequantized to obtain a floating-point approximation of the transmitted model object.

Ignoring clipping for notational simplicity, dequantization of an integer value *q* is given by

(19) $$\hat{x}=sq.$$

The payload encoding and decoding operations are lossless with respect to the quantized integer stream. Consequently, any difference between the original floating-point update and its reconstructed value originates from the quantization stage rather than from the integer or entropy coder.

For the running example, decoding recovers the unsigned stream:

[2, 3, 0],

which is mapped back to the signed quantized stream

[1, −2, 0].

Using s=0.01, dequantization yields

$$\begin{array}{cc} {\hat{\Delta w}}_{k}^{t} & =0.01\cdot [1,\;-2,\;0]\\ & =[0.01,\;-0.02,\;0.00]. \end{array}$$

For delta transmission, the reconstructed local model is therefore

(20) $${\hat{w}}_{k}^{t+1}=w^{t}+{\hat{\Delta w}}_{k}^{t}.$$

For the running example,

$${\hat{w}}_{k}^{t+1}=\left[0.50,\;-0.20,\;0.10\right]+\left[0.01,\;-0.02,\;0.00\right]=\left[0.51,\;-0.22,\;0.10\right].$$

Reconstruction is exact in this example because all delta values are representable at the selected quantization scale.

In addition to the encoded payload, FEDCODE accounts for the metadata required for reconstruction, including tensor shapes, quantization parameters, coder parameters, and codebooks when applicable. For each communication round, the framework records mean per-client uplink and server-object downlink payload sizes, compression rates, and encoding and decoding times. These quantities are subsequently used as communication and processing metrics for comparing the evaluated compression methods. Together, these references distinguish the impact of serialization, numerical approximation, and payload coding. The coder families described next are implementations of the payload-coding stage used in this evaluation. Additional coders can be incorporated at the same stage without altering the surrounding federated learning (FL) training, transmission, reconstruction, or measurement processes.

## 6. Compression Methods

This section outlines the compression methods evaluated in FEDCODE. All these methods utilize the common update-compression pipeline introduced in Section 5. Each method takes a tensor-valued transmitted object, which can either be a full parameter state or a delta update. This object is then transformed into the format required by the chosen compression method before constructing the payload. For the integer-coding methods considered in this study, the transmitted values are quantized, mapped to an unsigned integer stream, and then encoded using the selected payload coder.

The outlined methods represent a specific set of compression strategies used in this study rather than a fixed set of methods supported by FEDCODE. The framework separates update construction, numerical transformation, payload coding, decoding, and communication accounting, allowing alternative methods to be introduced at the corresponding stage of the pipeline while retaining the same FL execution and evaluation procedure. This design enables FEDCODE to function not only as an evaluation framework for the methods studied here but also as an experimental environment for developing and comparing new communication-reduction techniques.

Let

$$u=[u_{1},u_{2},\ldots ,u_{N}]$$

denote the unsigned integer stream that results from quantization and the conversion from signed to unsigned integers for the integer-coding methods. Payload coding is lossless with respect to its quantized input representation. Therefore, any approximation error occurs before payload coding, due to quantization and scale selection. This separation allows the experiments to distinguish between numerical approximation errors and lossless payload reduction.

### 6.1. Reference and Competitive Baseline Methods

Two internal reference methods provide comparison points for distinguishing the effects of numerical approximation and payload coding. The uncompressed baseline transmits the tensor-valued object without quantization or entropy coding. The serialized size of this uncompressed transmitted object is used as the reference when reporting compression rates. Because the serialized representation includes serialization and representation metadata, its size should not be interpreted as the storage size of the underlying floating-point tensor values alone, such as 4N bytes for *N* FP32 values. Instead, it provides an implementation-level communication reference that is measured using the same payload-level byte accounting applied to the evaluated compression methods.

For a compression method producing a payload of size $B_{\mathrm{method}}$, the reported compression rate is defined relative to the serialized uncompressed baseline $B_{\mathrm{baseline}}$ as

(21) $$\mathrm{CR}=100\left(1-\frac{B_{\mathrm{method}}}{B_{\mathrm{baseline}}}\right).$$

Here, $B_{\mathrm{baseline}}$ denotes the serialized size of the corresponding uncompressed transmitted object. Consequently, the reported compression rate quantifies reduction relative to FEDCODE’s serialized uncompressed baseline rather than relative to the storage size of the floating-point tensor values alone.

The quantization-only method employs fixed-scale quantization and tensor packing, without using variable-length integers or entropy coding. This approach isolates the impact of reduced numerical precision, separate from the additional reductions achieved through lossless coding of the quantized representation. By comparing a coded method to the uncompressed baseline, we can assess the combined reduction resulting from numerical approximation and payload coding. In contrast, comparing it to the quantization-only reference highlights the additional reduction that comes from coding the quantized integer stream.

To enhance our internal references, we assess three well-established methods for reducing communication in federated learning (FL): FedPAQ [24], FedBAT [50], and SparsyFed [64]. They represent complementary strategies: quantization, training-aware binarization, and sparsification with pruning of communicated updates.

Since FedBAT and SparsyFed alter the local training process in addition to modifying the transmitted representations, we treat them as complete FL methods during our evaluation alongside competitive baselines. To ensure a controlled comparison, we evaluate all three methods using a standardized experimental protocol defined in Section 7. This includes the same datasets, models, client sampling strategies, communication rounds, and local training settings. As a result, we interpret the findings as matched-protocol comparisons rather than precise reproductions of the original studies.

Collectively, the uncompressed and quantization-only references allow us to isolate the effects of numerical approximation and payload coding within FEDCODE, while FedPAQ, FedBAT, and SparsyFed offer an end-to-end comparison with established approaches for reducing communication in federated learning.

### 6.2. Universal Integer Codes

Universal integer codes are fixed prefix codes that do not require a learned symbol distribution, a transmitted codebook, or prior knowledge of symbol probabilities [65]. Since Elias codes are defined for positive integers, each unsigned symbol is shifted by one:

$$x_{i}=u_{i}+1.$$

Thus, a zero-valued quantized symbol $u_{i}=0$ is encoded as $x_{i}=1$.

Elias-γ coding represents a positive integer *x* by encoding its order of magnitude followed by its binary representation. If

$$m=\lfloor \log_{2}x\rfloor ,$$

then the codeword length is

$$l_{\gamma }\left(x\right)=2m+1.$$

Elias-δ coding first encodes the bit-length of *x* using Elias-γ, followed by the remaining lower-order bits of *x*. Let

$$b=\lfloor \log_{2}x\rfloor +1$$

be the bit-length of *x*. Then, its codeword length is

$$l_{\delta }\left(x\right)=l_{\gamma }\left(b\right)+b-1=2\left\lfloor \log_{2}b\right\rfloor +1+b-1=b+2\left\lfloor \log_{2}b\right\rfloor .$$

Elias-γ is well suited to small positive integers, while Elias-δ becomes more efficient for sufficiently large values because the overhead used to describe the integer magnitude grows more slowly. Both methods are simple, codebook-free, and parameter-free. Their variable-length codewords are concatenated at the bit level, and the mapping from integer values to codewords is fixed rather than adapted to the observed update distribution.

### 6.3. Golomb–Rice Coding

Golomb–Rice coding is a parameterized prefix-code family for nonnegative integers and is well suited to distributions in which small values occur more frequently than large ones [66,67]. Rice coding is the power-of-two specialization of Golomb coding; in the variant used by FEDCODE, the divisor is

$$M=2^{k},$$

which allows quotient and remainder computation using bit shifts and masks.

For a symbol *u*, Golomb–Rice coding computes

$$q=\left\lfloor \frac{u}{2^{k}}\right\rfloor ,\;r=u\;\mathrm{mod}\;2^{k}.$$

The quotient *q* is represented in unary form with a terminating bit, while the remainder *r* is represented using exactly *k* bits. The resulting codeword length is

$$l_{\mathrm{GR}}\left(u;k\right)=\left\lfloor \frac{u}{2^{k}}\right\rfloor +1+k.$$

The parameter *k* controls the trade-off between the unary quotient and the fixed-length remainder: increasing *k* reduces the quotient for larger symbols but allocates more remainder bits to every encoded symbol. In the static FEDCODE method, *k* remains fixed during the simulation, so coding efficiency depends on how well the selected value matches the distribution of the quantized integer stream.

### 6.4. Exponential-Golomb Coding

Exponential-Golomb coding is a parameterized prefix-code family for nonnegative integers and is used, for example, in the video coding standard H.264/AVC [69]. Unlike Golomb–Rice coding, whose fixed-*k* codeword length contains a unary quotient that grows approximately linearly with the symbol magnitude, Exponential-Golomb coding partitions the nonnegative integers into ranges whose sizes grow exponentially. Consequently, its codeword length grows logarithmically with the symbol magnitude, making occasional large symbols less costly than under a fixed-*k* Golomb–Rice code.

For order-*k* Exponential-Golomb coding and symbol *u*, the encoder forms

$$c=u+2^{k},$$

and writes n−k leading zeros followed by the (n+1)-bit binary representation of *c*, where

$$n=\lfloor \log_{2}c\rfloor .$$

The resulting codeword length is

$$l_{\mathrm{EG}}\left(u;k\right)=2\left\lfloor \log_{2}\left(u+2^{k}\right)\right\rfloor -k+1.$$

For k=0, this is equivalent to Elias-γ coding applied to u+1. Increasing *k* allocates more bits to the lower-order part of the representation while reducing the prefix length for larger symbols. The choice of *k* therefore determines how coding efficiency is distributed between small and larger symbol values. FEDCODE includes both Golomb–Rice and Exponential-Golomb coding because their codeword lengths grow differently with symbol magnitude and can therefore respond differently to the distribution of quantized model updates.

### 6.5. LEB128 and LEB$\left(2^{p}\right)$ Varint Coding

Variable-length integer coders represent an integer using a variable number of continuation groups. Standard unsigned LEB128 uses seven payload bits and one continuation bit in each byte and is used in formats such as DWARF and WebAssembly [70,71]. Values representable by seven payload bits require one byte, while larger values require additional bytes. The byte-aligned representation is simple and widely used, although its minimum codeword length of one byte can make it less bit-efficient than bit-level codes for streams containing many small integers.

FEDCODE additionally uses LEB$\left(2^{p}\right)$ as a tunable continuation-based generalization of this representation. Each group contains *p* payload bits and one continuation bit, so its payload field can represent $2^{p}$ possible values. Standard LEB128 corresponds to p=7 with byte-aligned 8-bit groups. For p<7, FEDCODE concatenates the (p+1)-bit groups directly in a bitstream rather than aligning each group to a byte. The notation LEB$\left(2^{p}\right)$ refers to the $2^{p}$ values representable by the payload portion of each group.

For a nonzero symbol *u*, the number of groups required with payload width *p* is

$$g_{p}\left(u\right)=\left\lceil \frac{\lfloor \log_{2}u\rfloor +1}{p}\right\rceil ,$$

with $g_{p}\left(0\right)=1$. The codeword length of standard byte-aligned LEB128 is therefore

$$l_{\mathrm{LEB}128}\left(u\right)=8g_{7}\left(u\right),$$

whereas the bit-packed LEB$\left(2^{p}\right)$ representation used by FEDCODE has codeword length

$$l_{\mathrm{LEB}(2^{p})}\left(u;p\right)=\left(p+1\right)g_{p}\left(u\right).$$

The parameter *p* determines a trade-off between group width and the number of groups required to represent a symbol. Smaller values of *p* produce shorter individual groups and can encode very small symbols using fewer bits, but larger symbols may require more continuation groups. Larger values of *p* carry more payload bits per group and therefore require fewer groups as symbol magnitudes increase. FEDCODE evaluates multiple payload widths to study this trade-off and, in its adaptive variant, selects a representation based on the observed quantized integer stream.

### 6.6. Huffman Coding

Huffman coding is included as a distribution-sensitive entropy coder [72]. Unlike the preceding integer codes, whose mappings from integer values to codewords are fixed for a given parameter setting, Huffman coding constructs a codebook from the empirical symbol frequencies of the current payload. More frequent symbols receive shorter codewords, while less frequent symbols receive longer codewords. This is attractive for quantized FL updates because delta updates and quantization can produce highly nonuniform symbol distributions, with many zero and small-magnitude values.

For an alphabet A with symbol probabilities p(a), the average Huffman codeword length is

$${\bar{l}}_{H}=\sum_{a\in A}p\left(a\right)l_{H}\left(a\right),$$

where $l_{H}\left(a\right)$ is the codeword length assigned to symbol *a*. The Shannon entropy of the symbol distribution is [83]

$$H\left(A\right)=-\sum_{a\in A}p\left(a\right)\log_{2}p\left(a\right).$$

For a binary Huffman code constructed for a source distribution, the average symbol-code length satisfies

$$H\left(A\right)\leq {\bar{l}}_{H}<H\left(A\right)+1.$$

Thus, Huffman coding adapts its codeword assignment to the observed symbol distribution and provides an average symbol-code length close to that distribution’s entropy. This relationship concerns the encoded symbol stream itself and does not include the additional cost of transmitting the codebook or other payload metadata.

In FEDCODE, Huffman coding is implemented as a static per-payload coder. The codebook is constructed from the empirical frequencies of the quantized symbols in the current transmitted payload and remains fixed while that payload is encoded and decoded. An additional end-of-stream symbol is included for decoding. Because the codebook and end-of-stream symbol are stored as metadata, the evaluation includes their communication overhead, along with codebook construction and decoding cost.

### 6.7. Adaptive Coder-Parameter Selection

Static coder parameters are simple, but they can be suboptimal because the distribution of quantized model updates can vary across datasets, clients, model states, and communication rounds. FEDCODE therefore includes adaptive variants of selected parameterized coders. The adaptive mechanisms are implemented within the same modular compression pipeline, allowing parameter-selection rules or alternative adaptive strategies to be introduced without modifying the surrounding FL training and communication workflow.

For the adaptive coder variants evaluated in this work, FEDCODE selects the coder parameter that minimizes the total symbol-code length of the current quantized integer payload:

(22) $$\theta_{c}^{★}=\arg\min_{\theta \in \Theta_{c}}\sum_{i=1}^{N}l_{c}\left(u_{i};\theta \right),$$

where *c* denotes the selected coder, $\Theta_{c}$ is its candidate parameter set, and $l_{c}\left(u_{i};\theta \right)$ is the codeword length assigned to symbol $u_{i}$ under parameter θ. Here, θ corresponds to the Rice parameter *k*, the Exponential-Golomb order *k*, or the LEB$\left(2^{p}\right)$ payload width *p*.

The optimization in Equation (22) is coder-aware because it evaluates candidate parameters using the code-length function of the coder that will encode the payload. The selected parameter is stored in the payload metadata required for decoding, and FEDCODE maintains the corresponding per-client adaptive state to support reproducible and resumable simulations. The optimization targets the coded symbol stream itself; metadata overhead and the computational cost of parameter selection are accounted for separately in the end-to-end evaluation.

### 6.8. Coder-Aware Adaptive Delta Scaling (CAADS)

A key methodological contribution of this work is the CAADS method for communication-efficient federated learning (FL). This method dynamically selects the quantization scale for each client update based on the update’s characteristics and the coding cost of the chosen payload coder. Unlike the adaptive coder-parameter selection described in Section 6.7, which optimizes the lossless representation of an already quantized integer stream, the proposed method adapts the numerical representation before coding.

The method is implemented as an adaptive stage of the FEDCODE compression pipeline and is independent of both the FL optimization procedure and a particular payload coder. Update statistics first provide a scale proposal, which is then refined using a coder-specific estimate of the resulting payload cost.

Let

$$\Delta ={\left\{\Delta_{i}\right\}}_{i=1}^{N}$$

denote the parameter-delta update transmitted by one client, where *N* is the number of scalar values across all transmitted trainable parameter tensors. FEDCODE maintains one adaptive scale *s* per client. Smaller scales preserve finer numerical changes but produce integers with larger magnitudes, whereas larger scales map more small updates to zero and concentrate the integer distribution around zero.

For a scale *s*, a value $\Delta_{i}$ rounds to zero when

$$|\Delta_{i}|<\frac{s}{2}.$$

The corresponding quantization-induced zero fraction is

(23) $$\rho_{0}\left(s\right)=\frac{1}{N}\sum_{i=1}^{N}I\left(|\Delta_{i}|<\frac{s}{2}\right).$$

The target zero fraction $\rho_{\mathrm{target}}$ provides a control signal for quantization aggressiveness. Because update magnitudes may vary across clients and rounds, the method also uses the root-mean-square update magnitude

(24) $$d=\sqrt{\frac{1}{N}\sum_{i=1}^{N}\Delta_{i}^{2}}$$

as a reference for the scale of the current update.

The zero-fraction and magnitude signals are combined into the update-informed proposal

(25) $$s_{\mathrm{prop}}=\mathrm{clip}\left(\left(1-\lambda \right)\left[\left(1-\eta \right)s+\eta s\exp\left(\rho_{\mathrm{target}}-\rho_{0}\left(s\right)\right)\right]+\lambda \alpha d,s_{\min},s_{\max}\right),$$

where η controls the adaptation rate, λ controls the influence of the RMS reference, α scales the RMS term, and $\left[s_{\min},s_{\max}\right]$ defines the admissible range. The previous client scale provides temporal continuity, while the remaining terms make the proposal responsive to the current update.

The proposal alone does not capture the communication cost of the resulting integer distribution. The method therefore performs a coder-aware local search around $s_{\mathrm{prop}}$. Candidate scales are defined as

(26) $$s_{m}=\mathrm{clip}\left(ms_{\mathrm{prop}},s_{\min},s_{\max}\right),\;m\in M,$$

where M is a predefined set of multiplicative search factors.

Each candidate produces a quantized integer representation

$$u\left(s_{m}\right)=\left[u_{1}\left(s_{m}\right),u_{2}\left(s_{m}\right),\ldots ,u_{N}\left(s_{m}\right)\right].$$

For coder *c*, its estimated symbol-code length is

(27) $$L_{c}\left(s_{m}\right)=\sum_{i=1}^{N}l_{c}\left(u_{i}\left(s_{m}\right)\right),$$

where $l_{c}\left(\cdot \right)$ denotes the coder-specific code-length function.

Minimizing code length alone could favor an excessively coarse scale. Candidate scales are therefore restricted by

$$\rho_{0}\left(s_{m}\right)\leq \rho_{\mathrm{target}}+\epsilon_{\rho },$$

where $\epsilon_{\rho }$ is the allowed tolerance above the target zero fraction. The final scale is selected as

(28) $$s^{★}=\arg\min_{s_{m}}L_{c}\left(s_{m}\right)\;\mathrm{subject}\;\mathrm{to}\;\rho_{0}\left(s_{m}\right)\leq \rho_{\mathrm{target}}+\epsilon_{\rho }.$$

Thus, update statistics determine the operating region, while coder-aware evaluation selects the transmitted representation with the lowest estimated coding cost subject to the zero-fraction constraint.

To limit computational overhead, FEDCODE estimates candidate code lengths on a subset of the update instead of fully encoding every candidate. The sampled fraction is controlled by coder_aware_sample_frac, and coder_aware_max_samples bounds the number of evaluated values. In the current implementation, at most 5% of update entries are used, capped at 250,000 values. Full encoding is performed only after the final scale has been selected. Algorithm 1 summarizes the procedure.
**Algorithm 1** Coder-Aware Adaptive Delta Scaling (CAADS)**Require:** 
Floating-point delta update Δ, previous client scale *s*, target zero fraction $\rho_{\mathrm{target}}$, adaptation rate η, magnitude weight λ, RMS scaling factor α, scale bounds $\left[s_{\min},s_{\max}\right]$, candidate multipliers M, zero-fraction tolerance $\epsilon_{\rho }$, coder *c***Ensure:** 
Selected delta scale $s^{★}$  1:Estimate $\rho_{0}\left(s\right)$  2:Compute the RMS update magnitude$$d\leftarrow \sqrt{\frac{1}{N}\sum_{i=1}^{N}\Delta_{i}^{2}}$$  3:Compute the update-informed proposal$$s_{\mathrm{prop}}\leftarrow \mathrm{clip}\left(\left(1-\lambda \right)\left[\left(1-\eta \right)s+\eta s\exp\left(\rho_{\mathrm{target}}-\rho_{0}\left(s\right)\right)\right]+\lambda \alpha d,s_{\min},s_{\max}\right)$$  4:Initialize $s^{★}\leftarrow s_{\mathrm{prop}}$ and $L^{★}\leftarrow \infty$  5:**for all** m∈M **do**  6:    $s_{m}\leftarrow \mathrm{clip}\left(ms_{\mathrm{prop}},s_{\min},s_{\max}\right)$  7:    Estimate $\rho_{0}\left(s_{m}\right)$  8:    **if** $\rho_{0}\left(s_{m}\right)\leq \rho_{\mathrm{target}}+\epsilon_{\rho }$ **then**  9:        Quantize a sampled subset of Δ using $s_{m}$10:        Map the quantized values to coder input symbols $u(s_{m})$11:        Estimate $L_{c}\left(s_{m}\right)$12:        **if** $L_{c}\left(s_{m}\right)<L^{★}$ **then**13:           $s^{★}\leftarrow s_{m}$14:           $L^{★}\leftarrow L_{c}\left(s_{m}\right)$15:        **end if**16:    **end if**17:**end for**18:**return** $s^{★}$

The proposed method differs from coder-parameter adaptation in its approach. While coder-parameter selection optimizes the lossless coding of a fixed quantized representation, the proposed method adapts the quantized representation itself based on the current update and its expected coding cost. Consequently, its effectiveness is evaluated comprehensively, considering predictive performance, transmitted payload size, metadata overhead, and the costs associated with encoding and decoding. Because the adaptive quantizer may introduce bias, Appendix A analyzes quantization error propagation over repeated rounds of FedAvg under Non-IID data conditions. It also derives sufficient conditions for convergence to a bounded neighborhood of a first-order stationary point.

## 7. Experimental Setup

This section outlines the methodology for evaluating the compression pipeline introduced in Section 5. We conduct the experiments using the FEDCODE framework described in Section 4, which provides a standardized environment for configuring compression pipelines, running federated learning (FL) experiments, and collecting measurements of communication, model quality, and runtime.

In addition to established methods for quantization, integer coding, and entropy coding, this study also evaluates CAADS. By implementing CAADS as a component of the FEDCODE pipeline, we enable a direct comparison with static compression techniques and coder-parameter adaptation while maintaining consistent FL conditions and measurement procedures.

The experiments examine how different compression-pipeline choices affect three primary outputs: transmitted data volume, model quality, and computational overhead. The following subsections define the research questions, experimental inputs, datasets and models, federated training protocol, compression configurations, evaluation procedure, measured outputs, execution environment, and reproducibility measures.

An experiment denotes one complete FL run. Experiments sharing the same FL configuration while varying one or more compression-related factors form an evaluation scenario.

### 7.1. Research Questions

This study considers four research questions:

**RQ1**  **(Communication efficiency): How do the choices made at different stages of the compression pipeline affect the transmitted data volume?**
  We examine how model-update representation, quantization, and lossless payload coding affect payload size. We pay special attention to how the representation and quantization stages shape the distribution of encoded values and influence the effectiveness of various integer and entropy coders.
**RQ2**  **(Model-quality impact): How do lossy compression choices affect the resulting model quality?**
  We evaluate how quantization precision and delta-scale selection affect the learning process and the final model quality. This evaluation characterizes the trade-off between compressibility and preserving important update information. Lossless payload coding is not considered a source of model degradation, as it accurately reconstructs the quantized representation.
**RQ3**  **(Adaptive compression): What advantages do adaptive compression mechanisms provide relative to corresponding static configurations, and under what conditions?**
  We study both coder-parameter adaptation and CAADS. The former selects coding parameters for the current quantized representation, whereas CAADS modifies the quantized representation using update statistics and coder-specific estimated code length, subject to a zero-fraction safeguard. We assess communication benefits alongside the associated computational and model-quality costs.
**RQ4**  **(Practical trade-offs): What trade-offs arise between transmitted data volume, computational overhead, and model quality from different compression pipeline configurations?**
  We consider communication, runtime, and model quality measurements together to identify optimal operating points under various communication and computational constraints.

### 7.2. Experimental Design and Study Inputs

Each experiment follows the synchronous Federated Learning (FL) process defined in Section 3 and is characterized by two configurations: the FL configuration and the compression-pipeline configuration.

The FL configuration defines the learning problem and training procedure. It includes the dataset, model architecture, client partition, total client population, number of participating clients per round, number of communication rounds, local epochs, batch size, learning rate, optimizer, aggregation rule, client-sampling procedure, and random seed. These parameters remain constant throughout a single evaluation scenario.

The compression-pipeline configuration details the factors being investigated. This includes the model-update representation, active pipeline stages, quantization configuration, and the payload coder along with its parameters, as well as any adaptive parameter-selection mechanism. One or more of these factors can be varied while keeping the FL configuration fixed.

This separation between FL configuration and compression-pipeline configuration allows for a clear assessment of how compression choices affect transmitted data volume, model quality, computational overhead, and, when relevant, the statistics of the encoded representation.

### 7.3. Datasets and Models

The study combines standard benchmark datasets with simulated client partitions and federated benchmarks with native user-level partitions. MNIST and CIFAR-10 provide controlled evaluation scenarios, while FEMNIST and Sent140 from LEAF introduce native client partitions and greater client heterogeneity [16,84,85].

For MNIST and CIFAR-10, the training data are divided into seeded IID client partitions of approximately equal size. FEMNIST and Sent140 retain the native LEAF user-level partitioning. Each dataset is paired with a model from the FEDCODE model registry, which validates dataset–model combinations and records the selected model identifier in the resolved experiment configuration. The evaluated datasets, tasks, models, and partition types are summarized in Table 2.

### 7.4. Federated Training Protocol

Table 3 summarizes the resolved federated training configuration used for each dataset. Compression configurations compared within the same evaluation scenario and random seed use the same client population, client-participation schedule, local-training configuration, model initialization, and aggregation procedure.

All available clients participate in every round for MNIST and CIFAR-10, as the number of clients in the population equals the number of clients selected in each round. For FEMNIST and Sent140, ten clients are sampled uniformly without replacement in each communication round from populations of 100 and 18,582–18,940 clients, respectively. The Sent140 population size depends on the random seed. Sampling is repeated in subsequent rounds, allowing a client to participate in more than one round. The client schedule is determined by the random seed and the round index, and the same schedule is reused for all compression configurations within an evaluation scenario for a given random seed.

At the beginning of each communication round, every selected client loads the same current version of the global model. This ensures that all clients start their local training from the same model for that round. The local training data is shuffled using a deterministic random generator that is specific to both the client and the round. Instead of discarding the final incomplete mini-batch, we retain it. Consequently, if a client’s local dataset has fewer samples than the configured batch size, that client will be trained using one smaller mini-batch per local epoch.

For the LEAF datasets, clients that do not satisfy the configured minimum local dataset requirements are excluded before the client pool is constructed. The reported experiments require at least three local training samples and one local test sample per retained user. No additional oversampling, duplication, or padding of small client datasets is performed.

A new optimizer is instantiated for every client participation. Optimizer state is therefore local to one local-training invocation and is reset between client participations. In particular, SGD momentum buffers and Adam moment estimates are not retained across communication rounds.

Only trainable tensors returned by the model’s named-parameter interface are included in the transmitted full- or delta-update representation. Registered non-trainable buffers are not transmitted or aggregated. The evaluated models do not use BatchNorm layers with client-specific running statistics: the MNIST model uses LayerNorm, the CIFAR-10 model uses GroupNorm, and the FEMNIST and Sent140 models contain no communicated BatchNorm state.

After decompression, the server applies sample-weighted FedAvg using the number of local training samples of each participating client. In full-update experiments, the reconstructed local parameter states are averaged directly. In delta-update experiments, the reconstructed client deltas are averaged using the same sample weights and added to the current global model, as defined in Section 3. Because the simulated MNIST and CIFAR-10 partitions are approximately equal in size, their sample-weighted aggregation is close to simple averaging; the same sample-weighted rule is nevertheless used consistently for all reported datasets.

### 7.5. Compression-Pipeline Configurations and Adaptive Methods

A compression-pipeline configuration specifies the model-update representation, active compression stages, methods implementing those stages, and their parameters. The pipeline itself is defined in Section 5.

Both full-update and delta-update representations are evaluated, as defined in Section 3. Because they produce different numerical distributions, update representation is treated as a compression-related input.

Four general classes of configurations are evaluated:

1. *Raw transmission*, in which the update is serialized without quantization or payload coding, providing the reference transmitted data volume.
2. *Quantization-only transmission*, in which the update is quantized without variable-length payload coding, isolating the effects of quantization.
3. *Static payload coding*, in which a lossless integer or entropy coder is applied without adaptive quantization-scale selection or coder-parameter search.
4. *Adaptive compression*, in which compression parameters are selected or adjusted according to the current model-update representation.

The principal quantization inputs are the quantization precision and, for delta updates, the delta scale *s*. These parameters affect both the numerical approximation and the symbol distribution supplied to the payload coder.

FEDCODE supports the lossless payload coders Elias-γ, Elias-δ, Golomb–Rice, Exponential-Golomb, LEB($2^{p}$), and Huffman coding. Because these coders operate losslessly on the quantized representation, they affect payload size and coding cost but introduce no additional numerical approximation.

For parameterized coders, static configurations use fixed parameters, whereas coder-parameter adaptation selects the coder parameter from the current quantized representation. This changes the lossless encoding of a fixed symbol stream without modifying the quantized values. Huffman coding constructs a codebook from the symbol frequencies; this is distinct from the coder-parameter adaptation evaluated for parameterized integer coders.

For the coder-parameter experiments, static reference configurations are selected through coarse sweeps over k∈{0,…,7} for Golomb–Rice and Exponential-Golomb and p∈{1,…,7} for LEB$\left(2^{p}\right)$. For each coder and update representation, the parameter minimizing the mean combined uplink–downlink payload is selected. The resulting delta-update references use k=0 and p=1, whereas the full-update references use k=4 and p=3. Values above the tested ranges are not considered because their minimum codeword length exceeds 8 bits per symbol and therefore cannot improve on the underlying 8-bit quantized representation at the symbol-code level. Adaptive variants instead select the coder parameter from the current quantized representation using the coder-specific code-length objective defined in Section 6.

The second adaptive mechanism is CAADS, defined in Section 6.8. Unlike coder-parameter adaptation, CAADS adjusts the quantization scale and therefore changes the integer-symbol distribution presented to the downstream coder. It combines update-statistics-based scale adaptation with coder-specific code-length evaluation, subject to a zero-fraction safeguard.

All configurations use the same FEDCODE interfaces for quantization, payload coding, FL execution, runtime measurement, and reproducibility.

For the competitive FL-compression baselines, method-specific configurations were selected following the published algorithms and available reference implementations within a matched experimental protocol. FedPAQ applies stochastic norm-based quantization with s=10, where *s* denotes the FedPAQ quantization parameter rather than the FEDCODE delta scale, to client model updates. FedBAT uses ϕ=0.5 and ρ=6, with binarization-aware local training applied to convolutional and linear weight updates, while biases and other auxiliary parameters are communicated densely in FP32. SparsyFed uses power reparameterization with β=1.25, adaptive activation sparsification, and global Top-*K* pruning of convolutional and linear pseudo-gradients at a target sparsity of 95%; biases and other auxiliary parameters remain dense. We use SparsyFed’s sparse-delta communication interpretation with the fixed dataset-specific learning rates reported in Table 3. For Sent140, the otherwise zero-initialized linear weights are initialized once using Xavier uniform initialization to avoid zero gradients under power reparameterization.

All three methods use the same dataset, model, client partition, sampled clients in each round, number of communication rounds, local epochs, batch size, sample-weighted aggregation rule, and random seeds as the corresponding FEDCODE comparison. Communication costs are computed from the serialized payloads and include encoding metadata and densely transported auxiliary parameters.

### 7.6. Evaluation Procedure and Experiment Groups

The evaluation is organized into five experiment groups, each varying a specific subset of compression inputs while keeping the corresponding FL configuration fixed.

First, we assess static configurations on MNIST and CIFAR-10 datasets. This evaluation includes raw transmission, quantization-only transmission, and static lossless coding for both full and delta updates. Additionally, we compare these methods with FedPAQ, FedBAT, and SparsyFed using the matched protocol outlined in Section 7.5.

Second, we analyze the effects of delta representation and quantization scale selection by measuring symbol distribution, payload size, and model quality. The comparison of quantization precision assesses 8-bit and 16-bit delta updates at a scale of s=0.01. An evaluation of the sensitivity of the 8-bit Huffman delta scale is conducted across various values of s∈{0.0003,0.001,0.005,0.01,0.05,0.1}. The primary static 8-bit delta configurations are set to s=0.01 for the MNIST and CIFAR-10 datasets, s=0.05 for FEMNIST, and s=0.0001 for Sent140.

Third, we evaluate adaptive configurations, including coder-parameter adaptation and CAADS. For CAADS, MNIST is evaluated at $z_{f}\in \left\{0.80,0.94\right\}$, while CIFAR-10 is evaluated at $z_{f}\in \left\{0.80,0.94,0.96\right\}$. A nested MNIST component ablation progressively introduces zero-fraction feedback, update-magnitude drift, and coder-aware candidate-scale refinement. Within each target setting, the compared variants retain the same FL configuration, random seeds, initial delta scale, and Huffman coding pipeline.

Fourth, we evaluate selected static and adaptive configurations along with competitive baselines on FEMNIST and Sent140. This evaluation uses native user-level partitions and partial client participation. The adaptive LEAF experiments are conducted with $z_{f}=0.94$.

Fifth, an edge-device replay experiment evaluates selected compression configurations on Raspberry Pi 3B and Raspberry Pi 5 hardware using MNIST and FEMNIST updates, with CIFAR-10 evaluated only on Raspberry Pi 5 only due to its larger update size.

Encoding and decoding times are collected throughout all experiment groups and reported with communication and model-quality measurements. Interpretation of the results with respect to the research questions is presented in Section 9.

### 7.7. Measured Outputs

#### 7.7.1. Transmitted Data Volume and Representation Statistics

Communication efficiency is measured using the mean serialized uplink payload per participating client, the serialized server-side downlink object size, and their reductions relative to the corresponding raw representation. The compression rate is defined as

(29) $$R_{\mathrm{comp}}=100\left(1-\frac{B_{\mathrm{compressed}}}{B_{\mathrm{raw}}}\right),$$

where $B_{\mathrm{compressed}}$ is the payload produced by the selected pipeline and $B_{\mathrm{raw}}$ is the corresponding raw payload.

For lossless coding, reduction relative to the quantization-only representation is also reported, separating coding savings from quantization effects. Payload sizes include reconstruction metadata such as tensor shapes, quantization and coder parameters, and Huffman codebooks. Competitive baselines also include sparse indices and densely communicated auxiliary tensors where applicable. In analytical lossless-suite runs, payload sizes are calculated rather than serialized.

The reported values follow the communication model in Section 3: uplink is reported as the mean payload per participating client, while the serialized server-side downlink object is counted once and may be multiplied by the number of selected clients when a unicast estimate is required.

For communication–runtime operating-point comparisons, total bidirectional representation-level communication is computed as the sum of the mean per-client uplink payload and the corresponding server-object downlink payload. Total communication reduction is evaluated relative to the equivalent raw bidirectional representation.

FEDCODE records symbol counts, zero fraction, and codeword-length statistics (mean, percentiles, maximum). These link update representation, quantization, and adaptive scale selection to coding efficiency and data volume.

Representation-level entropy and fitted-distribution statistics are estimated from pooled collected deltas. In contrast, zero fractions reported for the delta-scale experiments are measured within individual runs and then summarized across seeds. The pooled and per-run zero-fraction estimates therefore need not coincide exactly.

#### 7.7.2. Model Quality

Model quality is evaluated using classification accuracy and loss to assess the effects of lossy compression, particularly quantization precision and delta-scale selection. Lossless coders reconstruct the quantized representation exactly and are therefore evaluated through payload size and computational overhead, whereas coder-aware scale selection can affect model quality by changing the quantized values.

#### 7.7.3. Computational Overhead

Computational overhead is characterized using client-side encoding, server-side decoding, downlink encoding, and total round time. Adaptive scale-selection operations outside codec timing contribute only to total round time, which also includes local training and other round-level operations. Runtime comparisons are conducted only when the corresponding codecs are executed under matched hardware and software conditions.

For communication–runtime operating-point comparisons, mean round codec time combines the measured per-client uplink encoding and decoding time with the corresponding server-side downlink encoding time. Adaptive scale-selection operations outside the codec itself are excluded from this codec-time metric and are reflected only in total round time.

For communication–runtime scatter plots, a configuration is treated as Pareto-nondominated when no other measured configuration achieves at least as large a communication reduction and at most as large a codec time, with at least one of the two inequalities being strict. The reported Pareto frontier connects these nondominated measured operating points.

### 7.8. Execution Environments and Edge-Device Replay

The main federated-learning experiments were executed on Windows 11 Pro 25H2 (Build 26200) using an AMD Ryzen 9 9900X CPU, 64 GB of RAM, and an NVIDIA GeForce RTX 5070 Ti GPU with 16 GB of VRAM. The software environment used Python 3.14.2, PyTorch 2.9.1, and CUDA 12.8. Runtime measurements from these experiments are interpreted comparatively within this common desktop environment.

To complement the desktop measurements, selected FEDCODE compression configurations were replayed on a Raspberry Pi 5 Model B Rev. 1.1 and a Raspberry Pi 3 Model B Rev. 1.2. Both devices used Debian GNU/Linux 13 (trixie), Linux kernel 6.18.34+rpt-rpi-2712, and Python 3.13.5. The Raspberry Pi 5 uses a quad-core ARM Cortex-A76 processor at up to 2.4 GHz, while the Raspberry Pi 3B uses a Broadcom BCM2837 quad-core 64-bit ARM Cortex-A53 processor at 1.2 GHz with 1 GB of RAM. Previously collected model updates were passed through the same compression and decompression pipeline to isolate codec overhead on edge hardware. Each selected Raspberry Pi replay configuration is measured over 10 repeated codec executions. Server-side values reported alongside the replay results are taken from the corresponding desktop measurements in the main experiments and serve as the desktop reference.

The replay measurements are additionally used to estimate the bandwidth at which the reduction in transmission time produced by compression equals its additional codec cost. For method *m* relative to reference configuration *r*, the break-even bandwidth is

(30) $$R_{\mathrm{break},m}=\frac{8(B_{r}-B_{m})}{T_{\mathrm{codec},m}-T_{\mathrm{codec},r}},$$

where $B_{m}$ and $B_{r}$ are the transmitted payload sizes in bytes and $T_{\mathrm{codec}}=T_{\mathrm{enc}}+T_{\mathrm{dec}}$ is expressed in seconds. The factor 8 converts bytes to bits, and the resulting rate is reported in Mbit/s. For quantization, raw transmission is used as the reference; for Huffman configurations, the corresponding 8-bit quantization configuration is used.

For a specified effective bandwidth *R*, the corresponding communication-plus-codec time saving is

(31) $$S_{m}\left(R\right)=\frac{8(B_{r}-B_{m})}{R}-\left(T_{\mathrm{codec},m}-T_{\mathrm{codec},r}\right).$$

When evaluating $S_{m}\left(R\right)$, the reported bandwidth is converted from Mbit/s to bit/s. Positive $S_{m}\left(R\right)$ indicates that the reduction in transmission time exceeds the additional codec cost. Savings are evaluated at effective symmetric bandwidths of 1, 5, 15, 25, and 100 Mbit/s.

### 7.9. Reproducibility

Unless otherwise stated, each configuration is evaluated across 10 random seeds. Absolute communication, runtime, and model-quality measurements are reported as the mean and sample standard deviation across seeds. Model-quality differences between configurations are evaluated using same-seed paired differences with two-sided 95% Student-*t* confidence intervals.

Each FEDCODE run stores its resolved configuration and records round- and run-level model-quality, communication, runtime, and representation metrics. Competitive baselines additionally store method-specific parameters. Checkpoints retain the model, completed round, measurement history, adaptive state, and configuration, allowing interrupted runs to resume without resetting compression state.

The source code, experiment scripts, resolved run configurations, and analysis scripts used for the reported experiments are provided in the FEDCODE repository.

## 8. Results

Results follow the evaluation sequence defined in Section 7.6, with communication, model-quality, and runtime metrics reported jointly. Unless otherwise stated, values are rounded to two decimal places, with additional precision used where informative.

### 8.1. Static Compression and Competitive Baselines

This subsection compares raw transmission, 8-bit quantization, static lossless payload coding, and competitive FL-compression baselines on MNIST and CIFAR-10. Delta and full update representations are evaluated where applicable. The MNIST and CIFAR-10 experiments use 100 and 50 communication rounds, respectively.

**Figure 4:**
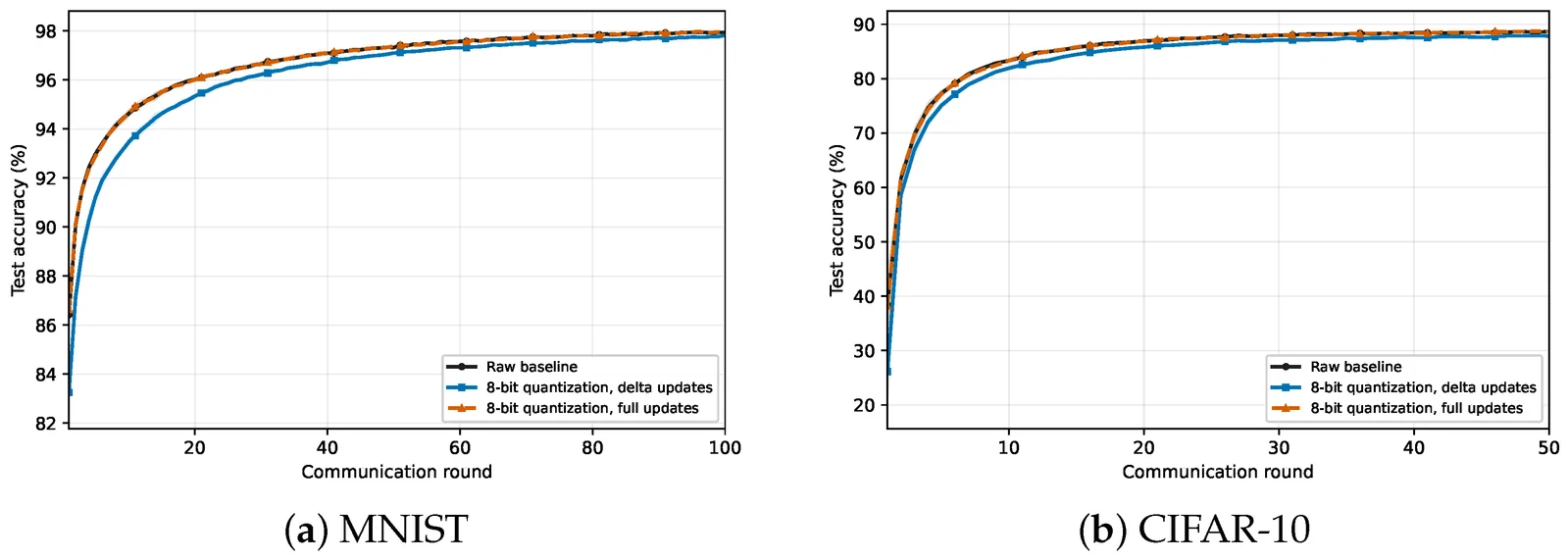
Test accuracy over communication rounds for raw transmission and 8-bit quantization with delta and full updates on MNIST and CIFAR-10. Curves show the mean across 10 random seeds, with shaded regions indicating one sample standard deviation.

Table 4 and Table 5 report communication, codec-runtime, and final-model-quality measurements, while Figure 4 shows the corresponding test-accuracy trajectories.

Across both datasets, 8-bit quantization reduces the raw payload by approximately 75%. Subsequent lossless coding provides substantially larger reductions for delta updates than for full updates. On MNIST, the strongest static delta configurations reduce both communication directions by approximately 96%, compared with about 80% for full updates. On CIFAR-10, the corresponding reductions are approximately 97% and 81%.

Among the static delta-update coders, Golomb–Rice and Huffman produce nearly identical payload sizes on both datasets, while Huffman has lower measured encoding and decoding times. Because payload coding is lossless, these methods retain the model quality of the corresponding quantized trajectory. The competitive baselines exhibit different communication–quality trade-offs. FedPAQ and FedBAT strongly reduce the uplink but retain an essentially dense downlink, whereas SparsyFed reduces both uplink and downlink through sparse communication. All three baselines show larger final-accuracy differences from the raw reference than the static FEDCODE delta-coding configurations.

**Figure 5:**
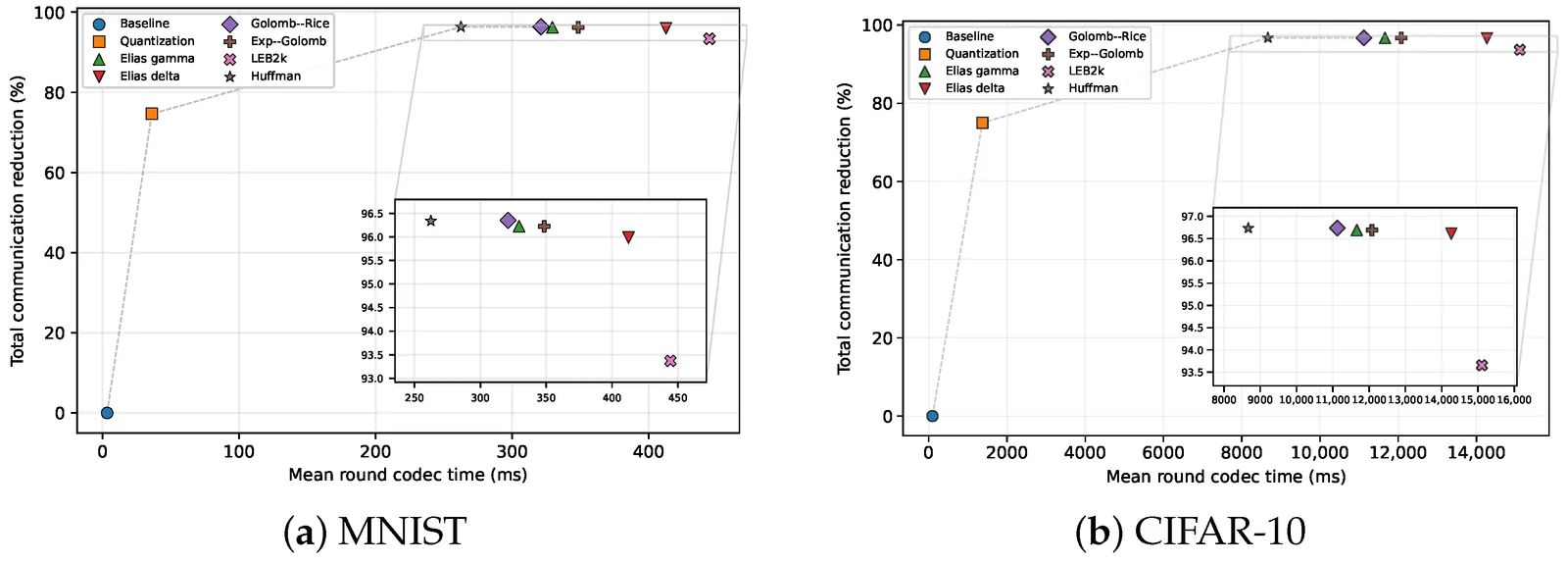
Total bidirectional communication reduction versus mean codec time for static FEDCODE delta-update configurations on MNIST and CIFAR-10. Values are averaged across 10 seeds.

Figure 5 summarizes the measured communication–runtime operating points for the static FEDCODE delta-update configurations. Golomb–Rice and Huffman provide nearly identical total communication reduction on both datasets, while Huffman has lower measured codec time in the evaluated implementation.

### 8.2. Delta-Scale and Encoded-Representation Measurements

This subsection examines the effects of quantization precision and delta scale on communication and model quality for MNIST and CIFAR-10. We first compare 8-bit and 16-bit quantized delta updates at s=0.01, followed by an 8-bit delta-scale sweep with Huffman payload coding. The reported measurements include uplink payload, final-model accuracy, quantization-induced zero fraction, and mean codeword length. Separate representation measurements at s=0.01 additionally characterize symbol entropy and the delta distribution. All configurations are evaluated across 10 random seeds.

Table 6 shows that 8-bit quantization approximately halves the quantized-delta payload relative to 16-bit quantization. On MNIST, the payload decreases from 480.54 to 242.67 KiB while mean final accuracy differs by 0.02 percentage points. On CIFAR-10, the payload decreases from 18032.77 to 9020.85 KiB while mean final accuracy differs by 0.19 percentage points.

Table 7 and Table 8 report the 8-bit delta-scale sweep with Huffman payload coding. Increasing *s* quantizes a larger fraction of the update to zero, reducing the mean codeword length and encoded payload. The final column reports the paired final-accuracy difference relative to s=0.01 for the same seed.

Both datasets show the same communication–quality trade-off. On MNIST, increasing *s* from 0.0003 to 0.01 reduces the uplink from 168.49 to 37.46 KiB. Relative to s=0.01, the paired final-accuracy differences at s=0.0003, 0.001, and 0.005 are +0.17, +0.14, and +0.14 percentage points, respectively. The corresponding pointwise confidence intervals exclude zero.

Further increases in scale yield much smaller payload reductions. On MNIST, s=0.05 reduces the payload to 30.98 KiB but lowers final accuracy by 9.81 percentage points relative to s=0.01. At s=0.1, the payload decreases by only another 0.14 KiB while the paired accuracy difference reaches −21.17 percentage points.

On CIFAR-10, increasing *s* from 0.0003 to 0.01 reduces the uplink from 5023.77 to 1206.93 KiB. Relative to s=0.01, the paired final-accuracy differences at s=0.0003, 0.001, and 0.005 are +0.62, +0.67, and +0.38 percentage points, respectively, with pointwise confidence intervals that exclude zero. At s=0.05, the payload decreases to 1129.88 KiB while final accuracy decreases by 8.14 percentage points relative to s=0.01. Increasing *s* to 0.1 reduces the payload by only another 1.26 KiB while the paired accuracy difference reaches −21.53 percentage points.

The zero-fraction and codeword-length measurements show the same saturation of the encoded representation at the largest scales. On MNIST, the zero fraction increases from 0.7186 at s=0.01 to 0.9875 and 0.9983 at s=0.05 and s=0.1, while the corresponding mean Huffman codeword length decreases from 1.2196 to 1.0053 and 1.0007 bits. On CIFAR-10, the zero fraction increases from 0.7718 to 0.9751 and 0.9949, while the mean codeword length decreases from 1.0694 to 1.0012 and 1.0001 bits.

**Figure 6:**
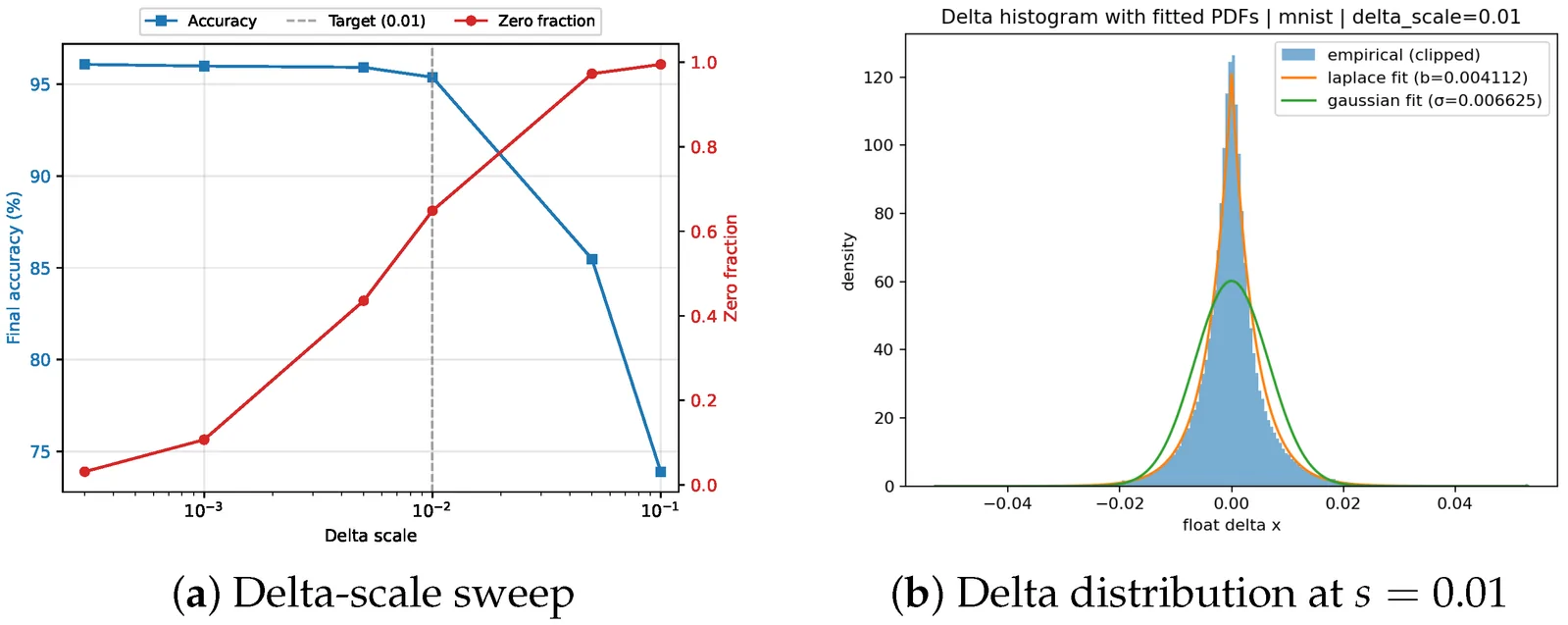
MNIST delta-scale and representation measurements. Panel (**a**) shows final accuracy and zero fraction across delta scales, with s=0.01 marked as reference; panel (**b**) shows the delta distribution at s=0.01 with Gaussian and Laplace fits.

**Figure 7:**
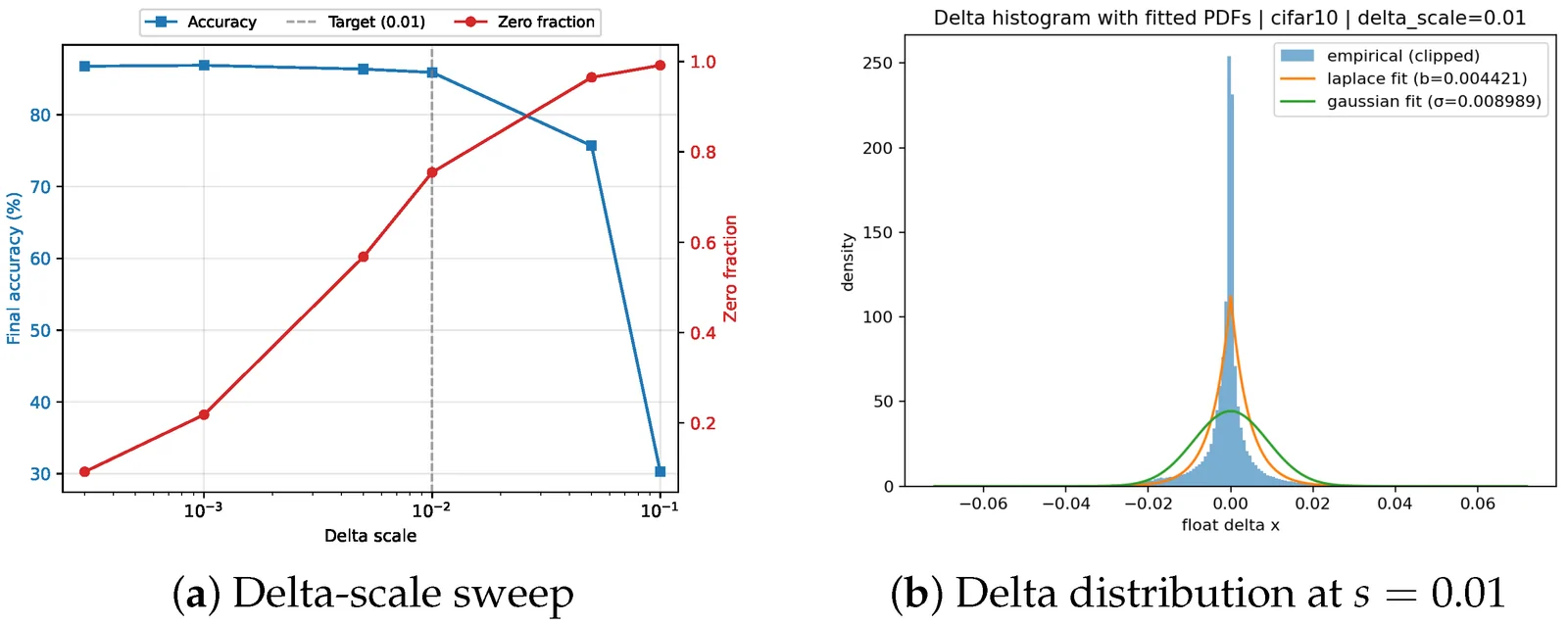
CIFAR-10 delta-scale and representation measurements. Panel (**a**) shows final accuracy and zero fraction across delta scales, with s=0.01 marked as reference; panel (**b**) shows the delta distribution at s=0.01 with Gaussian and Laplace fits.

Figure 6 and Figure 7 visualize the effect of delta scale on final model accuracy and the quantization-induced zero fraction, together with the corresponding delta distributions. In each figure, panel (a) shows mean final accuracy and zero fraction across the evaluated delta scales, with error bars indicating one sample standard deviation across 10 seeds and s=0.01 marked as the reference scale. Panel (b) shows the floating-point delta distribution at s=0.01 together with fitted Gaussian and Laplace densities.

The representation measurements at s=0.01 further characterize the distributions underlying the coding results. For MNIST, the zero fraction is $\rho_{0}=0.6487$, with empirical, Laplace-based, Gaussian-based, and nonzero-symbol entropies of 1.6145, 1.6440, 2.0443, and 1.9338 bits/symbol, respectively. For CIFAR-10, the corresponding zero fraction is $\rho_{0}=0.7547$, with empirical, Laplace-based, Gaussian-based, and nonzero-symbol entropies of 1.3791, 1.4024, 1.9636, and 2.3457 bits/symbol, respectively.

### 8.3. Adaptive Compression Measurements

This subsection reports the adaptive-compression experiments on MNIST and CIFAR-10. We first evaluate adaptive coder-parameter selection, followed by CAADS, a nested component ablation, and the round-wise communication–runtime measurements.

#### 8.3.1. Coder-Parameter Adaptation

Table 9 and Table 10 compare adaptive coder-parameter selection with the tuned static references for delta and full updates. Because coder-parameter selection changes only the lossless representation of the same quantized update, the comparison focuses on payload size and codec runtime. In the tables, Δ denotes adaptive minus static, so negative values indicate lower payload or runtime for the adaptive configuration.

For MNIST delta updates, adaptive selection closely reproduces the tuned static configurations: mean uplink differences are only 0.03–0.04 KiB with similarly small downlink differences. For full updates, all adaptive variants slightly reduce payload, with the largest reduction being 0.63 KiB for the LEB2k downlink. Runtime differences are also small except for full-update LEB2k, where adaptation adds approximately 30 ms of encoding and 18 ms of decoding time.

The CIFAR-10 delta-update results mirror MNIST: adaptive selection closely matches the tuned static parameters, with uplink differences below 0.3 KiB and downlink differences below 0.05 KiB. It adds some runtime for Golomb–Rice and Exponential-Golomb while leaving communication essentially unchanged.

For full updates, adaptation yields modest payload reductions for Golomb–Rice and Exponential-Golomb and a clearer improvement for LEB2k. Relative to fixed p=3, adaptive LEB2k reduces mean uplink and downlink payloads by 129.02 KiB and 132.55 KiB, respectively, while also reducing encoding and decoding time.

Across both datasets, adaptive coder selection closely matches the tuned static configurations for delta updates and provides larger payload reductions for selected full-update cases, most notably LEB2k on CIFAR-10.

#### 8.3.2. CAADS Results

The second adaptive experiment evaluates CAADS on MNIST and CIFAR-10. Payload and runtime differences are reported relative to the corresponding static coder, while model-quality differences are paired against static 8-bit delta quantization.

For MNIST, the CAADS results are reported in Table 11. At $z_{f}=0.80$ increases the encoded payload relative to the corresponding static coders, with mean uplink differences between +3.23 and +7.65 KiB. Final accuracy is 97.91–97.92%, corresponding to an approximately +0.10-percentage-point paired difference relative to static 8-bit quantization. At $z_{f}=0.94$, all coders reduce both uplink and downlink payload relative to their static counterparts; uplink differences range from −2.61 to −6.57 KiB. Final accuracy is 97.13%, with a paired difference of approximately −0.69 percentage points.

For CIFAR-10, the CAADS results are reported in Table 12. At $z_{f}=0.80$ produces larger encoded payloads than the corresponding static coders, with uplink differences from +277.64 to +602.35 KiB. Final accuracy is 88.62%, with a paired difference of +0.79 percentage points relative to static 8-bit quantization. At $z_{f}=0.94$, the payload differences are substantially smaller: uplink differences range from +17.65 to +45.84 KiB, while downlink differences range from +4.80 to +14.63 KiB. Final accuracy is 88.11% for Elias gamma and Elias delta and 87.96% for the remaining reported coders. At $z_{f}=0.96$, the adaptive payload is further reduced across all coders, with final accuracy between 87.63% and 87.66%.

#### 8.3.3. Component Ablation of CAADS

A nested component ablation on MNIST assesses the incremental effect of the mechanisms used by CAADS. Starting from zero-fraction feedback, the evaluation progressively adds update-magnitude drift and coder-aware candidate-scale refinement. A static Huffman configuration at s=0.005 is included as a non-adaptive reference.

At $z_{f}=0.80$, adding magnitude drift increases the mean uplink from 41.90 to 44.36 KiB and the downlink from 34.20 to 35.06 KiB, while final accuracy changes from 97.91% to 97.95%. Adding coder-aware refinement then reduces the uplink to 42.11 KiB and the downlink to 34.01 KiB, with a final accuracy of 97.87%.

At $z_{f}=0.94$, adding magnitude drift increases the uplink from 34.79 to 37.56 KiB and the downlink from 31.94 to 32.66 KiB, while final accuracy increases from 97.22% to 97.81%. Adding coder-aware refinement reduces the corresponding payloads to 33.68 and 31.29 KiB, respectively, with a final accuracy of 97.14%. Relative to the zero-fraction-only configuration, the complete method reduces mean uplink by 1.11 KiB and downlink by 0.65 KiB, while final accuracy differs by −0.08 percentage points.

#### 8.3.4. Adaptive Dynamics and Communication–Runtime Measurements

**Figure 8:**
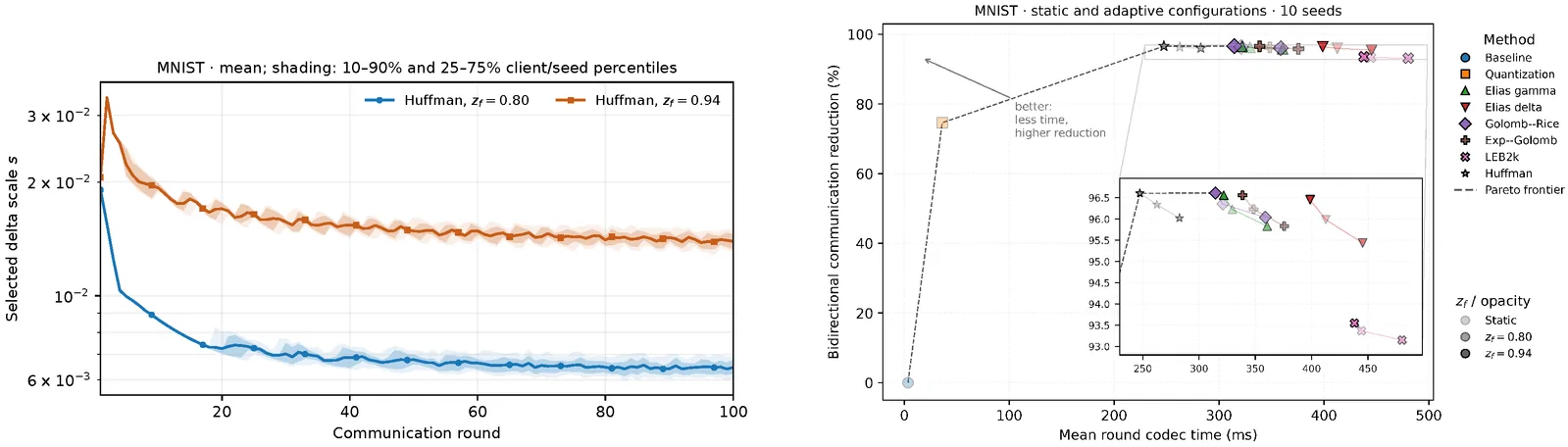
Adaptive compression measurements on MNIST. (**Left**): round-wise delta-scale values for the Huffman configuration. (**Right**): communication reduction versus mean round codec time across the evaluated adaptive configurations; the inset enlarges the high-reduction region and the dashed line indicates the measured Pareto frontier.

Figure 8 summarizes the dynamic behavior and communication–runtime characteristics of CAADS on MNIST. The selected delta scale changes most strongly during the early communication rounds and then gradually settles into a narrower range during later training. Most coded configurations achieve total communication reductions above 90%, while their mean round codec times vary substantially.

The measurements show that configurations with similar communication reductions can occupy different codec-runtime operating points. The corresponding payload, runtime, and final-accuracy measurements are reported in Table 11 and Table 13.

### 8.4. Federated Benchmark Evaluation on LEAF Datasets

This subsection evaluates static compression and CAADS on FEMNIST and Sent140 using the native LEAF user-level partitions. Both tasks are evaluated over 100 communication rounds using 10 random seeds. The adaptive evaluation considers $z_{f}=0.94$ together with the static compression configurations and the FedPAQ, FedBAT, and SparsyFed baselines. Table 14 and Table 15 summarize the resulting communication cost, codec runtime, and final model accuracy for FEMNIST and Sent140, respectively.

As shown in Table 14, the raw FEMNIST representation requires 1676.02 KiB in each direction and reaches 76.77±1.33% final accuracy. Static 8-bit quantization reduces both uplink and downlink payloads to 421.22 KiB, a 74.87% reduction, while reaching 75.99±0.98% accuracy. Its paired difference relative to the raw baseline is −0.78 percentage points, with a 95% confidence interval of [−1.91,+0.35].

Static lossless coding provides substantially larger communication reductions while retaining the 8-bit quantized model trajectory. For the Elias, Golomb, and Huffman coders, mean FEMNIST uplink payloads are approximately 58–62 KiB, corresponding to reductions of about 96.3%–96.6% relative to raw transmission. Golomb–Rice requires 57.73 KiB uplink and 54.32 KiB downlink, while Huffman requires 57.83 KiB and 54.37 KiB, respectively. LEB2k produces the larger coded representation at 108.49 KiB uplink and 105.89 KiB downlink.

The coder-aware adaptive configuration at $z_{f}=0.94$ reduces the transmitted representation slightly further for the Elias, Golomb, and Huffman coders. Mean uplink payloads range from 53.83 to 54.95 KiB, with final accuracy of 75.09±1.31%. The paired accuracy difference relative to the raw baseline is −1.68 percentage points, with a 95% confidence interval of [−2.85,−0.51].

The native federated-compression baselines in Table 14 exhibit different communication patterns. FedPAQ reduces the mean uplink to 261.91 KiB but retains a nearly full-size 1675.74 KiB downlink and reaches 57.40±4.91% final accuracy. FedBAT reduces the uplink to 53.96 KiB while retaining the same dense downlink and reaches 54.48±4.24%. SparsyFed reduces both directions to 129.09 KiB uplink and 169.91 KiB downlink, with a final accuracy of 5.50±0.24%.

Table 15 shows that the raw Sent140 representation requires 79.13 KiB in each direction and reaches 57.54±4.06% final accuracy. Static 8-bit quantization reaches 58.76±1.01%, corresponding to a paired difference of +1.22 percentage points relative to the raw baseline, with a 95% confidence interval of [−1.23,+3.67]. The separate uplink payload and codec timing measurements for this quantization run were not recorded.

Static lossless coding reduces the Sent140 representation substantially while retaining the 8-bit quantized model trajectory. Elias gamma, Elias delta, Golomb–Rice, and Exponential-Golomb require approximately 2.89–2.90 KiB per transmitted object, corresponding to reductions of about 96.3% relative to raw transmission. Huffman is similar at 2.95 KiB uplink and 2.93 KiB downlink, whereas LEB2k produces the larger coded representation of 5.35 KiB in each direction.

For the coder-aware adaptive configuration at $z_{f}=0.94$, the transmitted representation remains close to that of the corresponding static coders. Across the Elias, Golomb, and Huffman configurations, mean uplink payload is approximately 2.90–2.96 KiB, while final accuracy is 56.93±3.33%. Relative to the raw baseline, the paired difference is −0.61 percentage points, with a 95% confidence interval of [−1.24,+0.02].

Among the native federated-compression baselines in Table 15, FedPAQ reduces the uplink to 12.38 KiB while retaining a 78.84 KiB dense downlink and reaches 57.62±3.96% final accuracy. FedBAT reduces the uplink to 2.66 KiB with the same dense downlink and reaches 53.64±1.59%. SparsyFed yields the smallest transmitted representations, with 0.80 KiB uplink and 8.00 KiB downlink, and it reaches 50.86±2.68% final accuracy.

### 8.5. Edge-Device Replay Results

To provide context for the replay measurements obtained from the edge-devices, we compare the results from the Raspberry Pi with the server-side codec measurements discussed in the previous subsections. Table 16 presents the average total codec time, represented as $T_{\mathrm{codec}}=T_{\mathrm{enc}}+T_{\mathrm{dec}}$, along with the corresponding break-even bandwidth for 8-bit quantization, static Huffman coding, and the selected coder-aware Huffman configurations. Quantization is evaluated relative to raw transmission, while the Huffman configurations are assessed against their corresponding 8-bit quantized representation.

The platform measurements show a consistent ordering in codec execution time. For 8-bit quantization, the server break-even bandwidth is 305.59 Mbit/s on MNIST, 328.20 Mbit/s on FEMNIST, and 326.27 Mbit/s on CIFAR-10. The corresponding Raspberry Pi 5 values are 170.66, 126.61, and 193.96 Mbit/s, while the Raspberry Pi 3B reaches 23.29 Mbit/s on MNIST and 18.69 Mbit/s on FEMNIST.

For the Huffman configurations, the break-even bandwidths are lower on all three platforms. Across the evaluated configurations, they range from 10.38 to 16.20 Mbit/s on the server, 4.93 to 6.29 Mbit/s on the Raspberry Pi 5, and 0.66 to 0.82 Mbit/s on the Raspberry Pi 3B.

Table 17 reports the communication-plus-codec time savings defined in Equation (31) at the five representative bandwidths. To avoid duplicating every adaptive operating point, the table includes 8-bit quantization, static Huffman coding, and the representative $z_{f}=0.94$ coder-aware Huffman configuration.

At 1 Mbit/s, static and coder-aware Huffman coding have positive $S_{m}\left(R\right)$ values on the server and Raspberry Pi 5 for all evaluated datasets. At 5 Mbit/s, the corresponding values remain positive for all server measurements and for MNIST and CIFAR-10 on the Raspberry Pi 5, while static Huffman on FEMNIST is approximately at zero (−0.004 s). On the Raspberry Pi 3B, the Huffman configurations have negative $S_{m}\left(R\right)$ values at both 1 and 5 Mbit/s. For 8-bit quantization, $S_{m}\left(R\right)$ remains positive at all five reported bandwidths on the server and Raspberry Pi 5; on the Raspberry Pi 3B it is positive at 1, 5, and 15 Mbit/s and negative at 25 and 100 Mbit/s.

## 9. Discussion

This section interprets the measurements reported in Section 8 with respect to the four research questions defined in Section 7.1. Communication volume, model quality, and computational overhead are considered jointly, followed by broader observations, practical implications for edge federated learning, and the limitations of the study.

### 9.1. RQ1: Communication Efficiency

RQ1 asks how the choices made across the compression pipeline affect the volume of transmitted data. Quantization consistently achieves an initial reduction of about 75% for datasets such as MNIST, CIFAR-10, and FEMNIST. However, the additional benefit of lossless coding depends strongly on the update representation. For instance, Huffman coding reduces the volume of MNIST delta updates by approximately 96% compared to raw transmission, while it achieves about 80% reduction for full updates. A similar trend is observed with CIFAR-10.

The statistical properties of quantized delta updates explain this difference. These updates typically contain many low-magnitude values and a high proportion of zeros, making them far more amenable to variable-length coding than full updates. As the Huffman codeword length approaches one bit per symbol, further gains from delta-scale sweeps diminish. Compression efficiency therefore depends not only on the coding method but also on the symbol distribution produced by earlier representation and quantization stages.

The competitive baselines further underline the importance of bidirectional accounting. FedPAQ and FedBAT strongly reduce the client uplink while retaining dense downlinks in the evaluated configurations, whereas SparsyFed reduces traffic in both directions. Uplink reduction alone therefore gives an incomplete picture of the communication cost of an FL compression method.

#### Answer to RQ1

The largest reductions arise from combining delta updates, low-precision quantization, and lossless payload coding. Quantization provides the initial reduction, while the additional coding gain depends strongly on the resulting symbol distribution and is substantially larger for delta than for full updates.

### 9.2. RQ2: Model-Quality Impact

RQ2 examines how lossy compression choices impact model quality. In the evaluated pipeline, the main lossy decisions are quantization precision and delta-scale selection, while the subsequent integer and entropy coders precisely reconstruct the quantized representation.

In the main experiments with static delta updates, 8-bit quantization produced only small changes in final accuracy, and these changes varied by dataset. Relative to raw transmission, the paired differences are −0.12 percentage points for MNIST and −0.84 percentage points for CIFAR-10 (both with confidence intervals exclude zero). On FEMNIST and Sent140, the differences are −0.78 and +1.22 percentage points, respectively, with intervals that include zero. A direct comparison of 8-bit and 16-bit quantization shows only small differences in mean accuracy, while the 16-bit method approximately doubles the quantized payload.

Delta-scale selection has a markedly stronger effect. On both MNIST and CIFAR-10, moderate scales preserve model quality while reducing the coded payload. Aggressive settings, by contrast, drive the representation toward a near-one-bit-per-symbol coding floor and cause substantial accuracy loss. Beyond this threshold, further increases in zero concentration yield only minimal extra communication reduction relative to the accompanying drop in model quality.

The competitive baselines reinforce the same broader point. Under the matched protocol used in this study, strong communication reduction can be accompanied by substantially different model-quality outcomes. These measurements should not be interpreted as universal rankings of the methods, but they illustrate why communication efficiency should be evaluated jointly with preservation of the learning trajectory.

#### Answer to RQ2

At the evaluated operating points, 8-bit quantization substantially reduces payload with limited model-quality change relative to 16-bit quantization. Delta-scale selection is more consequential: moderate scales preserve useful update information, whereas overly aggressive scales provide diminishing communication gains at a substantial quality cost.

### 9.3. RQ3: Adaptive Compression

RQ3 investigates the benefits of adaptive compression compared to static configurations. The experiments differentiate between coder-parameter adaptation, which modifies only the lossless representation of a fixed quantized stream, and CAADS, which alters the quantized representation itself.

For delta updates, coder-parameter adaptation primarily acts as an automatic tuning mechanism. In experiments on MNIST and CIFAR-10, adaptive Golomb–Rice, Exponential-Golomb, and LEB2k closely match the payloads of optimized static configurations. When a suitable fixed parameter is already established, adaptation offers minimal additional reduction but eliminates the need to manually select that parameter.

For full model updates, the effect depends more on the coder and data representation. On MNIST, adaptive selection yields only small payload changes, while full-update LEB2k incurs additional runtime. On CIFAR-10, all three adaptive parameterized coders reduce payload, with the largest improvement for LEB2k, where mean uplink and downlink decrease by 129.02 and 132.55 KiB, respectively. Coder-parameter adaptation is therefore most useful when the favorable parameter varies with the update distribution rather than remaining fixed across the experiment.

CAADS changes the communication–quality operating point more directly. On MNIST, $z_{f}=0.80$ increases payload relative to the static coders but improves final accuracy by approximately 0.10 percentage points relative to static 8-bit quantization. At $z_{f}=0.94$, all evaluated coders reduce both uplink and downlink payload, while final accuracy decreases by approximately 0.69 percentage points. The target zero fraction therefore acts as an operating-point control rather than simply as a compression parameter.

Experiments on CIFAR-10 exhibit a similar mechanism but within a different useful range. At $z_{f}=0.80$, the adaptive payload sizes exceed the static references, and final accuracy improves. At $z_{f}=0.94$, the adaptive payloads remain close to or slightly above those of the static coders. However, at $z_{f}=0.96$, the adaptive payloads become smaller than their static counterparts, while the differences in final accuracy compared to 8-bit quantization are minimal, and their confidence intervals include zero. This indicates that the optimal target value depends on the specific dataset.

The nested ablation analysis offers deeper insights into the adaptive mechanism. At $z_{f}=0.94$, introducing update-magnitude drift increases the payload while improving accuracy from 97.22% to 97.81%. However, with coder-aware refinement, the payload drops below that of the zero-fraction-only configuration, with a final accuracy of 97.14%. This pattern suggests that the drift mechanism functions as a quality-preserving correction, while the coder-aware stage enhances communication efficiency. Nevertheless, the nested design does not allow us to isolate the individual effects of each component.

The round-wise scale trajectories further show that the adaptive controller changes its operating point during training. Larger adjustments occur early, followed by a narrower range in later rounds, which is consistent with the method responding to changes in the update distribution over the course of training.

The LEAF experiments further show that adaptive operating points do not transfer uniformly across tasks. On FEMNIST, $z_{f}=0.94$ produces a modest additional payload reduction but at the cost of a larger reduction in final accuracy, indicating that the increased zero concentration is obtained through more aggressive quantization of informative update components. On Sent140, the same target provides essentially no additional communication reduction relative to the static coders, suggesting that the resulting change in the quantized-symbol distribution does not translate into a smaller encoded representation. This also illustrates that zero fraction is only a proxy for compressibility: the achieved payload depends on the complete symbol distribution and the coder, while the model-quality effect depends on the task-specific update distribution. A fixed target should therefore not be expected to provide the same communication–quality balance across datasets, models, and client-partition structures.

#### Answer to RQ3

Coder-parameter adaptation is primarily useful as automatic tuning and can provide additional gains when the payload-efficient coder parameter varies with the update representation. CAADS can shift the trade-off between model quality and communication reduction, but the useful operating point is task-dependent and must account for acceptable model-quality and runtime costs.

### 9.4. RQ4: Practical Trade-Offs

RQ4 examines how transmitted data volume, computational overhead, and model quality interact in practical compression choices. The results show that the configuration with the smallest payload is not necessarily the fastest one once codec execution time is taken into account.

Quantization provides a large communication reduction at comparatively low codec cost. Although lossless coding can reduce the representation substantially—particularly for delta updates—it requires considerably more computation. Static results further show that coders with nearly identical payload sizes can differ noticeably in runtime; Huffman and Golomb–Rice achieve similar compression on MNIST and CIFAR-10, yet Huffman has shorter measured codec time in the evaluated implementation.

The edge replay clearly illustrates this trade-off. Quantization remains advantageous over a much wider bandwidth range than Huffman coding across all evaluated platforms. The break-even thresholds for employing additional coding decrease substantially as we move from server systems to Raspberry Pi 5 and further to Raspberry Pi 3B. This indicates that the effectiveness of an additional coding stage depends directly on the client’s computational capabilities.

The $S_{m}\left(R\right)$ measurements reinforce the same conclusion. At low bandwidth, the transmission time saved by Huffman coding can exceed its codec cost on the server and Raspberry Pi 5, whereas the same coding stage is already too expensive on Raspberry Pi 3B. Quantization remains beneficial over a broader range because its additional computational cost is much lower.

Adaptive compression introduces another layer of trade-offs, as CAADS can modify both the payload and model quality, while also adding computational tasks outside the codec-only timing used in break-even analysis. Consequently, end-to-end deployment decisions should factor in total round costs, rather than focusing solely on codec-level communication thresholds, especially when adaptive-selection overhead is significant.

#### Answer to RQ4

No single configuration simultaneously minimizes payload, computational cost, and model-quality change under all conditions. Quantization remains useful across a wide range of link rates, while additional lossless coding becomes attractive only when its transmission-time savings exceed the codec cost on the target device. Adaptive compression introduces a further quality–payload trade-off that should be chosen according to the intended deployment constraints.

### 9.5. Observations Beyond the Research Questions

In the experiments, the performance of compression is more closely tied to the representation given to the coder than to the coder itself. Factors such as update type, quantization precision, delta scale, dataset, and training state all affect the resulting symbol distribution. As a result, measured zero fractions, empirical entropies, and codeword lengths provide a valuable link between the numerical representation of updates and the final serialized output.

The comparison between FedPAQ, FedBAT, and SparsyFed highlights the differences in communication semantics among various FL-compression methods. Some configurations mainly reduce the client uplink, while others decrease both uplink and downlink traffic. Therefore, a comprehensive communication-aware comparison should specify what is transmitted in each direction and include the necessary metadata for reconstruction, rather than relying solely on a single compression ratio.

The experiments also demonstrate FEDCODE’s role as both an evaluation and development framework. The same modular workflow evaluates static compression, adaptive coder parameters, CAADS, representation diagnostics, runtime measurement, and edge-device replay, allowing comparison of their communication and model-quality effects under a common measurement procedure.

### 9.6. Practical Implications for Edge Federated Learning

The edge measurements indicate that compression decisions should be based on the effective application-level throughput available to a client rather than nominal physical-layer data rates, together with the associated codec costs. This distinction matters because practical wireless throughput often differs from nominal link rates due to protocol and implementation effects [86]. For example, on the Raspberry Pi 5, measurements indicate that 8-bit quantization has break-even bandwidths of approximately 127 to 194 Mbit/s, while the evaluated Huffman configurations reach their crossover point near 5 to 6 Mbit/s. In comparison, on the Raspberry Pi 3B, these corresponding ranges decrease to approximately 19 to 23 Mbit/s and below 1 Mbit/s, respectively.

These results show that adding a lossless coding stage can be beneficial for bandwidth-constrained links, but it may be counterproductive when using a slower processor, particularly when network throughput is higher. As an illustrative comparison, the mobile measurements from Ofcom referenced in this study reflect effective data rates of approximately 24.6, 15.2, and 2.7 Mbit/s for the respective 5G, 4G, and 3G scenarios [87]. Based on the simplifying assumptions applied in this analysis, Huffman coding on a Raspberry Pi 5 is primarily relevant for the lower throughput regime, while quantization remains advantageous across a significantly broader range of conditions.

These comparisons are illustrative rather than technology-specific predictions. Real FL links can be asymmetric and shared among clients, while protocol overhead, scheduling, retransmissions, and packet loss can change the effective throughput available to an update. Deployment-specific decisions should therefore combine measured payload and device runtime with the target environment’s observed uplink and downlink characteristics.

### 9.7. Limitations

The main FL experiments are simulation-based and run on a single desktop environment. In contrast, the Raspberry Pi experiments extend the runtime evaluation to two edge processors by replaying previously collected model updates. The replay measurements therefore characterize edge-device codec execution rather than complete on-device FL training. The desktop and Raspberry Pi measurements also use different measurement procedures and should be interpreted as platform-level references rather than strictly controlled processor benchmarks.

The break-even analysis focuses on representation-level communication and codec runtime. It does not explicitly model transport- and link-layer overhead, packetization, contention, retransmissions, or energy consumption. These factors can shift the practical crossover point but do not change the underlying criterion that communication savings must exceed the additional compression cost.

FedPAQ, FedBAT, and SparsyFed broaden the comparison beyond the dense quantization-and-coding pipeline considered in FEDCODE. They provide quantization-, binarization-, and sparsification-based reference points under the same federated protocol, although the comparison is not exhaustive. Their results should therefore be interpreted as matched-protocol evaluations rather than exact reproductions of the complete experimental settings used in the respective original studies.

CAADS assesses a limited number of nearby scale candidates by utilizing update statistics and code-length estimates specific to the coder. This approach enables practical local selection, but it does not necessarily optimize final accuracy, convergence speed, end-to-end latency, or energy consumption. The nested component ablation evaluates the mechanisms incrementally rather than testing every possible combination in isolation, which helps to interpret their combined effects without needing to separate each contribution.

The evaluated datasets and models span diverse federated learning settings. These include seeded independent and identically distributed (IID) partitions on MNIST and CIFAR-10, as well as user-level partitions with partial participation on FEMNIST and Sent140. While these scenarios provide controlled and varied testing environments, factors such as greater device diversity, fluctuating network conditions, nonstationary data, and larger model sizes may affect the performance outcomes. Therefore, validating these approaches in additional deployment settings is a key area for future research.

## 10. Conclusions

This paper presents FEDCODE, a modular, communication-aware framework for developing and evaluating communication-efficient model-update compression methods for federated learning. FEDCODE separates federated training from the representation and compression of transmitted model information, allowing researchers to vary update representation, quantization, payload coding, and adaptive mechanisms under a common, controlled experimental protocol. The framework provides bidirectional communication accounting, codec-runtime and model-quality measurements, encoded-representation statistics, reproducible experiment configuration, and checkpointing. It was further used to develop and evaluate the Coder-Aware Adaptive Delta Scaling (CAADS) method proposed in this work.

Experiments conducted on MNIST, CIFAR-10, and LEAF-based federated datasets demonstrate that communication efficiency is determined by the interaction of multiple compression-pipeline stages, including update representation, quantization scale, and payload coder. Using eight-bit quantization provides an initial reduction of approximately 75% relative to raw transmission, while combining quantization with delta-update representation and lightweight integer or entropy coding increases the reduction to approximately 96% in the main MNIST and CIFAR-10 experiments.

The additional coding gain is substantially smaller for full updates, confirming that coding effectiveness depends strongly on the symbol distribution produced by the preceding representation and quantization stages. Matched-protocol comparisons with FedPAQ, FedBAT, and SparsyFed further reveal favorable communication–quality trade-offs for the evaluated FEDCODE delta-coding configurations, as well as substantial differences among methods in their uplink and downlink behavior. These results reinforce the need for bidirectional rather than uplink-only communication accounting.

Measuring representations clarifies the trade-off between communication quality and efficiency. Increasing the delta scale increases the percentage of values quantized to zero, reducing both the average codeword length and the encoded payload. However, the communication benefits start to plateau as the representation approaches a near one-bit-per-symbol coding limit. Beyond this point, further increases in scale yield minimal additional payload reductions but can significantly degrade model quality. In contrast, the evaluated 8-bit and 16-bit quantization configurations yield similar final model quality, despite nearly a twofold difference in quantized payload. Since the subsequent integer and entropy coders are lossless, they maintain the learning trajectory of the corresponding quantized representation.

The two adaptive mechanisms act at different stages of the compression pipeline. Coder-parameter adaptation selects lossless coding parameters automatically; for delta updates, it generally matches rather than consistently outperforms well-tuned fixed settings. CAADS instead adjusts the quantization scale using update statistics and coder-specific code-length estimates. Its target zero fraction controls the trade-off between payload size and model quality: conservative settings better preserve quality, whereas more aggressive settings reduce payload at the cost of greater quality degradation.

The FEMNIST and Sent140 experiments extend previous observations to consider native user-level partitions, larger client populations, and partial client participation. The findings indicate that adaptive targets and operating points do not transfer uniformly across different datasets and update distributions. On Sent140, the same adaptive target performs worse because the increased zero fraction does not translate into a more compressible symbol distribution, so the additional quantization does not provide corresponding payload savings. Similarly, the competitive baselines show varying communication and quality behavior under the same protocol, highlighting the importance of evaluating payload reduction alongside model quality and communication flow.

The edge-device replay further indicates that byte reduction alone is not sufficient to determine practical compression benefit. The practical value of stronger payload coding therefore depends jointly on device capability and available network bandwidth. Compression choices should consequently be evaluated in terms of transmitted data volume, model quality, codec runtime, execution platform, and effective network throughput.

Overall, the findings support approaching communication-efficient FL as a pipeline-level communication–runtime–quality optimization problem rather than as payload minimization alone. FEDCODE offers a reproducible environment to expose and measure these interactions under controlled comparisons. At the same time, CAADS demonstrates that downstream coding characteristics can be incorporated directly into adaptive quantized-update representation selection. The optimal operating point depends on factors such as update distribution, learning task, desired model quality, device capability, codec cost, and communication environment.

Future work will extend the CAADS objective beyond estimated code length, to incorporate explicit model-quality, latency, energy, bandwidth, and device-resource constraints. Candidate selection could also exploit per-layer statistics, update history, and measured communication conditions. Further evaluation will consider larger models, additional federated datasets, heterogeneous devices, energy measurements, and real communication links, as well as hybrid policies that combine quantization, sparsification, entropy coding, and adaptive selection within the same framework.

## Figures and Tables

**Table 1:** Main extensions of the present journal article over the MIPRO 2026 conference paper.

Aspect	MIPRO 2026	Journal Version
Framework	Initial FEDCODE framework and proof-of-concept evaluation.	Extended framework with reproducible runs, detailed communication/runtime accounting, checkpointing, and additional federated datasets.
Compression methods	Static compression and adaptive entropy coders.	Extended coder evaluation and the proposed CAADS method.
Parameter analysis	Selected quantization and coder settings.	Full/delta-update evaluation, 8/16-bit quantization, delta-scale analysis, coder-parameter selection, and adaptive-method ablation.
Experimental scope	Preliminary single-seed MNIST and CIFAR-10 experiments with five clients.	Multi-seed MNIST/CIFAR-10 experiments, competitive FedPAQ, FedBAT, and SparsyFed baselines, and statistical analysis.
Federated heterogeneity	No native large-population Non-IID evaluation.	LEAF FEMNIST and Sent140 with native partitions, larger client pools, and partial participation.
Edge validation	Desktop/simulation runtime measurements only.	Raspberry Pi 3B and Raspberry Pi 5 edge-device replay measurements, including device-side codec latency, break-even effective bandwidth, and communication-plus-codec analysis.

**Table 2:** Datasets and model configurations considered in the reported experimental study.

Dataset	Task	Model	Partition
MNIST [84]	10-class digit classification	Improved MLP	IID
CIFAR-10 [85]	10-class image classification	VGG11-GN	IID
FEMNIST [16]	62-class character classification	CNN	Native users
Sent140 [16]	Binary sentiment classification	BoW logistic regression	Native users

**Table 3:** Federated training protocol used in the reported experiments. Here, *K* denotes the total client population available to the simulation. For Sent140, *K* varies across the ten random seeds.

Dataset	*K*	Clients/Round	Rounds	Local Epochs	Batch Size	Optimizer	Learning Rate
MNIST	5	5	100	1	5	SGD	0.01
CIFAR-10	5	5	50	5	5	SGD	0.01
FEMNIST	100	10	100	3	10	Adam	0.004
Sent140	18,582–18,940	10	100	1	32	SGD	0.0003

**Table 4:** Static compression and competitive FL-compression baselines on MNIST. Values are mean ± sample standard deviation across seeds 1–10; brackets show two-sided 95% Student-*t* confidence intervals for paired accuracy differences.

Method	Mean Up (KiB)	Up Red. (%)	Down Obj. (KiB)	Down Red. (%)	Enc. (ms)	Dec. (ms)	Final Acc. (%)	Δ Acc. (pp)
Raw reference								
Baseline	956.28 ± 0.00	0.00 ± 0.00	956.28 ± 0.00	0.00 ± 0.00	–	–	97.93 ± 0.05	+0.00 ± 0.00 [+0.00, +0.00]
Delta updates								
8b Quant.	242.67 ± 0.00	74.62 ± 0.00	242.67 ± 0.00	74.62 ± 0.00	18.33 ± 2.51	0.80 ± 0.20	97.81 ± 0.05	−0.12 ± 0.05 [−0.16, −0.08]
Elias gamma	39.16 ± 0.02	95.91 ± 0.00	32.98 ± 0.01	96.55 ± 0.00	131.94 ± 3.22	74.20 ± 2.39	97.81 ± 0.05	−0.12 ± 0.05 [−0.16, −0.08]
Elias delta	42.76 ± 0.03	95.53 ± 0.00	34.02 ± 0.02	96.44 ± 0.00	158.54 ± 4.19	108.48 ± 4.30	97.81 ± 0.05	−0.12 ± 0.05 [−0.16, −0.08]
Golomb–Rice (k=0)	37.31 ± 0.02	96.10 ± 0.00	32.48 ± 0.01	96.60 ± 0.00	137.87 ± 3.32	58.23 ± 1.74	97.81 ± 0.05	−0.12 ± 0.05 [−0.16, −0.08]
Exp-Golomb (k=0)	39.16 ± 0.02	95.90 ± 0.00	32.99 ± 0.01	96.55 ± 0.00	136.90 ± 3.27	84.01 ± 2.52	97.81 ± 0.05	−0.12 ± 0.05 [−0.16, −0.08]
LEB2k (p=1)	65.11 ± 0.01	93.19 ± 0.00	61.75 ± 0.00	93.54 ± 0.00	169.63 ± 4.23	114.09 ± 3.68	97.81 ± 0.05	−0.12 ± 0.05 [−0.16, −0.08]
Huffman	37.46 ± 0.02	96.08 ± 0.00	32.57 ± 0.01	96.59 ± 0.00	112.37 ± 2.71	48.12 ± 1.12	97.81 ± 0.05	−0.12 ± 0.05 [−0.16, −0.08]
Full updates								
8b Quant.	242.67 ± 0.00	74.62 ± 0.00	242.67 ± 0.00	74.62 ± 0.00	38.91 ± 51.01	13.27 ± 38.54	97.95 ± 0.05	+0.02 ± 0.07 [−0.03, +0.07]
Elias gamma	243.60 ± 3.12	74.53 ± 0.33	243.77 ± 3.14	74.51 ± 0.33	348.30 ± 83.16	279.09 ± 55.83	97.95 ± 0.05	+0.02 ± 0.07 [−0.03, +0.07]
Elias delta	242.19 ± 2.20	74.67 ± 0.23	242.31 ± 2.21	74.66 ± 0.23	393.42 ± 86.29	306.02 ± 49.74	97.95 ± 0.05	+0.02 ± 0.07 [−0.03, +0.07]
Golomb–Rice (k=4)	183.50 ± 1.60	80.81 ± 0.17	183.68 ± 1.61	80.79 ± 0.17	380.18 ± 98.42	236.78 ± 43.63	97.95 ± 0.05	+0.02 ± 0.07 [−0.03, +0.07]
Exp-Golomb (k=4)	190.68 ± 1.65	80.06 ± 0.17	190.79 ± 1.66	80.05 ± 0.17	320.28 ± 79.99	252.45 ± 53.99	97.95 ± 0.05	+0.02 ± 0.07 [−0.03, +0.07]
LEB2k (p=3)	219.66 ± 2.12	77.03 ± 0.22	219.83 ± 2.12	77.01 ± 0.22	334.77 ± 28.39	267.46 ± 21.91	97.95 ± 0.05	+0.05 ± 0.04 [+0.02, +0.08]
Huffman	186.57 ± 1.51	80.49 ± 0.16	186.63 ± 1.52	80.48 ± 0.16	416.39 ± 107.30	241.11 ± 45.37	97.95 ± 0.05	+0.02 ± 0.07 [−0.03, +0.07]
Native federated-compression baselines								
FedPAQ	149.44 ± 0.00	84.37 ± 0.00	956.00 ± 0.00	0.03 ± 0.00	110.50 ± 10.82	166.97 ± 17.76	97.45 ± 0.08	−0.48 ± 0.08 [−0.54, −0.43]
FedBAT	35.77 ± 0.00	96.26 ± 0.00	956.00 ± 0.00	0.03 ± 0.00	40.96 ± 4.13	43.28 ± 2.95	95.27 ± 0.10	−2.66 ± 0.09 [−2.73, −2.59]
SparsyFed	93.89 ± 3.44	90.18 ± 0.36	105.95 ± 0.00	88.92 ± 0.00	0.81 ± 0.11	0.36 ± 0.19	87.83 ± 0.44	−10.11 ± 0.47 [−10.44, −9.77]

**Table 5:** Static compression and competitive FL-compression baselines on CIFAR-10. Values are mean ± sample standard deviation across seeds 1–10; brackets show two-sided 95% Student-*t* confidence intervals for paired accuracy differences.

Method	Mean Up (KiB)	Up Red. (%)	Down Obj. (KiB)	Down Red. (%)	Enc. (ms)	Dec. (ms)	Final Acc. (%)	Δ Acc. (pp)
Raw reference								
Baseline	36,056.64 ± 0.00	0.00 ± 0.00	36,056.64 ± 0.00	0.00 ± 0.00	–	–	88.68 ± 0.25	+0.00 ± 0.00 [+0.00, +0.00]
Delta updates								
8b Quant.	9020.85 ± 0.00	74.98 ± 0.00	9020.85 ± 0.00	74.98 ± 0.00	671.38 ± 71.67	7.43 ± 0.46	87.84 ± 0.34	−0.84 ± 0.46 [−1.17, −0.51]
Elias gamma	1230.73 ± 1.28	96.59 ± 0.00	1154.92 ± 0.18	96.80 ± 0.00	4504.62 ± 170.06	2592.57 ± 172.27	87.84 ± 0.34	−0.84 ± 0.46 [−1.17, −0.51]
Elias delta	1275.50 ± 1.96	96.46 ± 0.01	1167.81 ± 0.27	96.76 ± 0.00	5255.33 ± 165.45	3690.17 ± 159.27	87.84 ± 0.34	−0.84 ± 0.46 [−1.17, −0.51]
Golomb–Rice (k=0)	1206.64 ± 0.93	96.65 ± 0.00	1148.13 ± 0.13	96.82 ± 0.00	4586.06 ± 156.15	2065.54 ± 173.07	87.84 ± 0.34	−0.84 ± 0.46 [−1.17, −0.51]
Exp-Golomb (k=0)	1230.74 ± 1.28	96.59 ± 0.00	1154.92 ± 0.18	96.80 ± 0.00	4635.94 ± 157.03	2902.05 ± 187.33	87.84 ± 0.34	−0.84 ± 0.46 [−1.17, −0.51]
LEB2k (p=1)	2308.28 ± 0.60	93.60 ± 0.00	2267.86 ± 0.08	93.71 ± 0.00	5668.21 ± 162.39	3872.39 ± 190.97	87.84 ± 0.34	−0.84 ± 0.46 [−1.17, −0.51]
Huffman	1206.83 ± 0.93	96.65 ± 0.00	1148.32 ± 0.13	96.82 ± 0.00	3577.77 ± 66.29	1645.91 ± 171.05	87.84 ± 0.34	−0.84 ± 0.46 [−1.17, −0.51]
Full updates								
8b Quant.	9020.69 ± 0.01	74.98 ± 0.00	9020.69 ± 0.01	74.98 ± 0.00	669.64 ± 51.72	4.62 ± 0.29	88.65 ± 0.24	−0.03 ± 0.31 [−0.25, +0.20]
Elias gamma	9421.51 ± 106.15	73.87 ± 0.29	9464.13 ± 108.74	73.75 ± 0.30	9772.91 ± 954.79	7765.06 ± 889.16	88.65 ± 0.24	−0.03 ± 0.31 [−0.25, +0.20]
Elias delta	9333.86 ± 70.72	74.11 ± 0.20	9364.67 ± 72.24	74.03 ± 0.20	10,958.82 ± 1047.28	8711.54 ± 948.39	88.65 ± 0.24	−0.03 ± 0.31 [−0.25, +0.20]
Golomb–Rice (k=4)	6815.21 ± 54.87	81.10 ± 0.15	6862.30 ± 56.42	80.97 ± 0.16	9994.18 ± 960.08	6386.18 ± 681.30	88.65 ± 0.24	−0.03 ± 0.31 [−0.25, +0.20]
Exp-Golomb (k=4)	7236.18 ± 54.87	79.93 ± 0.15	7266.04 ± 56.42	79.85 ± 0.16	8630.48 ± 806.51	6742.30 ± 712.21	88.65 ± 0.24	−0.03 ± 0.31 [−0.25, +0.20]
LEB2k (p=3)	8303.96 ± 53.71	76.97 ± 0.15	8338.99 ± 55.02	76.87 ± 0.15	10,982.70 ± 1017.01	9103.16 ± 982.44	88.65 ± 0.24	−0.03 ± 0.31 [−0.25, +0.20]
Huffman	6725.60 ± 65.41	81.35 ± 0.18	6744.44 ± 67.95	81.29 ± 0.19	11,484.66 ± 1147.84	6388.95 ± 508.40	88.65 ± 0.24	−0.03 ± 0.31 [−0.25, +0.20]
Native federated-compression baselines								
FedPAQ	5634.09 ± 0.00	84.37 ± 0.00	36,056.19 ± 0.01	0.00 ± 0.00	3857.13 ± 506.49	6194.15 ± 1210.28	82.27 ± 0.54	−6.41 ± 0.53 [−6.79, −6.03]
FedBAT	1149.46 ± 0.00	96.81 ± 0.00	36,056.16 ± 0.02	0.00 ± 0.00	1375.98 ± 193.38	1756.50 ± 373.38	86.28 ± 0.26	−2.41 ± 0.29 [−2.61, −2.20]
SparsyFed	3589.73 ± 25.25	90.04 ± 0.07	3647.30 ± 0.00	89.88 ± 0.00	14.80 ± 0.55	11.80 ± 0.41	83.90 ± 0.93	−4.80 ± 0.75 [−5.99, −3.61]

Uplink is mean payload per client; downlink is the encoded server payload. Reductions and Δ acc. are paired against the same-seed raw delta baseline. Enc./Dec. are mean per-client uplink codec times. Lossless coding retains the corresponding quantized accuracy.

**Table 6:** Comparison of 8-bit and 16-bit quantized delta updates on MNIST and CIFAR-10 using delta scale s=0.01. MNIST is evaluated over 100 communication rounds and CIFAR-10 over 50 rounds. Values are reported as mean ± sample standard deviation across 10 random seeds.

Dataset	Precision	Mean Uplink (KiB)	Accuracy (%)
MNIST	8-bit	242.67 ± 0.00	97.81 ± 0.05
MNIST	16-bit	480.54 ± 0.01	97.79 ± 0.08
CIFAR-10	8-bit	9020.85 ± 0.00	87.84 ± 0.34
CIFAR-10	16-bit	18,032.77 ± 0.01	88.03 ± 0.34

**Table 7:** Delta-scale sensitivity of 8-bit quantized delta updates with Huffman coding on MNIST over 100 communication rounds. Values are mean ± sample SD across seeds 1–10. Brackets give pointwise two-sided 95% Student-*t* confidence intervals for paired accuracy differences relative to s=0.01.

Delta Scale	Zero Frac. ($\rho_{0}$)	Mean CW (Bits)	Uplink (KiB)	Final Acc. (%)	Δ Acc. vs. s=0.01 (pp)
0.0003	0.0377 ± 0.0009	5.4897 ± 0.0026	168.49 ± 0.07	97.96 ± 0.07	+0.17 ± 0.13 [+0.08 ± +0.26]
0.001	0.1260 ± 0.0018	3.7744 ± 0.0012	114.78 ± 0.03	97.93 ± 0.07	+0.14 ± 0.07 [+0.09 ± +0.19]
0.005	0.5006 ± 0.0034	1.6470 ± 0.0015	50.36 ± 0.05	97.94 ± 0.06	+0.14 ± 0.12 [+0.06 ± +0.23]
0.01	0.7186 ± 0.0024	1.2196 ± 0.0006	37.46 ± 0.02	97.79 ± 0.08	–
0.05	0.9875 ± 0.0003	1.0053 ± 0.0001	30.98 ± 0.00	87.98 ± 0.59	−9.81 ± 0.61 [−10.24 ± −9.38]
0.1	0.9983 ± 0.0001	1.0007 ± 0.0000	30.84 ± 0.00	76.62 ± 2.66	−21.17 ± 2.68 [−23.09 ± −19.25]

**Table 8:** Delta-scale sensitivity of 8-bit quantized delta updates with Huffman coding on CIFAR-10 over 50 communication rounds. Values are mean ± sample SD across seeds 1–10. Brackets give pointwise two-sided 95% Student-*t* confidence intervals for paired accuracy differences relative to s=0.01.

Delta Scale	Zero Frac. ($\rho_{0}$)	Mean CW (Bits)	Uplink (KiB)	Final Acc. (%)	Δ Acc. vs. s=0.01 (pp)
0.0003	0.0988 ± 0.0018	4.4527 ± 0.0103	5023.77 ± 11.63	88.66 ± 0.24	+0.62 ± 0.38 [+0.35 ± +0.89]
0.001	0.2395 ± 0.0020	2.7329 ± 0.0121	3083.09 ± 13.65	88.70 ± 0.38	+0.67 ± 0.37 [+0.40 ± +0.93]
0.005	0.6010 ± 0.0018	1.2432 ± 0.0026	1402.94 ± 2.92	88.41 ± 0.34	+0.38 ± 0.33 [+0.14 ± +0.61]
0.01	0.7718 ± 0.0006	1.0694 ± 0.0006	1206.93 ± 0.64	88.03 ± 0.34	–
0.05	0.9751 ± 0.0006	1.0012 ± 0.0000	1129.88 ± 0.03	79.90 ± 0.45	−8.14 ± 0.32 [−8.36 ± −7.91]
0.1	0.9949 ± 0.0001	1.0001 ± 0.0000	1128.62 ± 0.00	66.50 ± 1.78	−21.53 ± 1.82 [−22.83 ± −20.23]

**Table 9:** MNIST coder-parameter adaptation relative to the selected static references. Adaptive payloads are reported as mean ± sample standard deviation across 10 random seeds.

Method	Static Ref.	Adaptive Up (KiB)	Δ Up (KiB)	Adaptive Down (KiB)	Δ Down (KiB)	$\Delta t_{\mathrm{enc}}$ (ms)	$\Delta t_{\mathrm{dec}}$ (ms)
Delta updates							
Adapt. Golomb–Rice	k=0	37.35 ± 0.02	+0.04	32.52 ± 0.01	+0.04	−1.80	+0.12
Adapt. Exp-Golomb	k=0	39.20 ± 0.02	+0.04	33.03 ± 0.01	+0.04	−0.69	−0.53
Adapt. LEB2k	p=1	65.14 ± 0.02	+0.03	61.79 ± 0.02	+0.04	+0.77	−2.99
Full updates							
Adapt. Golomb–Rice	k=4	183.15 ± 1.53	−0.35	183.23 ± 1.54	−0.45	−3.72	+0.41
Adapt. Exp-Golomb	k=4	190.61 ± 1.64	−0.07	190.68 ± 1.65	−0.11	−0.99	+0.23
Adapt. LEB2k	p=3	219.13 ± 1.81	−0.53	219.20 ± 1.80	−0.63	+30.28	+18.21

**Table 10:** CIFAR-10 coder-parameter adaptation relative to the selected static references. Adaptive payloads are reported as mean ± sample standard deviation across 10 random seeds.

Method	Static Ref.	Adaptive Up (KiB)	Δ Up (KiB)	Adaptive Down (KiB)	Δ Down (KiB)	$\Delta t_{\mathrm{enc}}$ (ms)	$\Delta t_{\mathrm{dec}}$ (ms)
Delta updates							
Adapt. Golomb–Rice	k=0	1206.82 ± 0.91	+0.18	1148.16 ± 0.13	+0.03	+84.51	+54.05
Adapt. Exp-Golomb	k=0	1230.97 ± 1.25	+0.23	1154.95 ± 0.17	+0.03	+112.35	+90.75
Adapt. LEB2k	p=1	2308.33 ± 0.59	+0.05	2267.82 ± 0.09	−0.04	+27.00	−17.92
Full updates							
Adapt. Golomb–Rice	k=4	6794.14 ± 54.40	−21.07	6817.09 ± 56.45	−45.21	−152.14	+7.64
Adapt. Exp-Golomb	k=4	7227.58 ± 54.72	−8.60	7250.24 ± 56.32	−15.80	−47.75	+31.63
Adapt. LEB2k	p=3	8174.94 ± 56.74	−129.02	8206.44 ± 59.46	−132.55	−2262.06	−1751.80

**Table 11:** CAADS on MNIST with 8-bit delta updates over 100 communication rounds. Values are mean ± sample SD across 10 seeds; accuracy differences are paired against static 8-bit quantization with two-sided 95% Student-*t* confidence intervals.

$z_{f}$	Method	Adaptive Up (KiB)	Δ Up (KiB)	Adaptive Down (KiB)	Δ Down (KiB)	$\Delta t_{\mathrm{enc}}$ (ms)	$\Delta t_{\mathrm{dec}}$ (ms)	Final Acc. (%)	Δ Acc. vs. 8b Quant. (pp)
0.80	Elias gamma	44.79 ± 0.04	+5.64	34.90 ± 0.03	+1.92	+13.88	+9.13	97.92 ± 0.06	+0.10 ± 0.08 [+0.05 ± +0.16]
0.80	Elias delta	50.42 ± 0.05	+7.65	36.90 ± 0.04	+2.88	+15.28	+8.81	97.92 ± 0.06	+0.10 ± 0.08 [+0.05 ± +0.16]
0.80	Golomb–Rice (k=0)	41.87 ± 0.04	+4.55	33.90 ± 0.02	+1.41	+17.14	+9.63	97.92 ± 0.06	+0.10 ± 0.08 [+0.05 ± +0.16]
0.80	Exp-Golomb (k=0)	44.80 ± 0.04	+5.64	34.91 ± 0.03	+1.92	+12.27	+8.03	97.92 ± 0.06	+0.10 ± 0.08 [+0.05 ± +0.16]
0.80	LEB2k (p=1)	68.34 ± 0.03	+3.23	62.69 ± 0.01	+0.94	+15.88	+12.40	97.92 ± 0.06	+0.10 ± 0.08 [+0.05 ± +0.16]
0.80	Huffman	42.08 ± 0.03	+4.62	34.00 ± 0.02	+1.43	+10.63	+6.26	97.91 ± 0.07	+0.10 ± 0.07 [+0.05 ± +0.16]
0.94	Elias gamma	34.45 ± 0.01	−4.71	31.36 ± 0.01	−1.63	−4.85	−1.40	97.13 ± 0.10	−0.69 ± 0.09 [−0.75 ± −0.62]
0.94	Elias delta	36.20 ± 0.02	−6.57	31.65 ± 0.01	−2.37	−7.91	−4.09	97.13 ± 0.10	−0.69 ± 0.09 [−0.75 ± −0.62]
0.94	Golomb–Rice (k=0)	33.57 ± 0.01	−3.74	31.22 ± 0.00	−1.26	−6.73	+0.39	97.13 ± 0.10	−0.69 ± 0.09 [−0.75 ± −0.62]
0.94	Exp-Golomb (k=0)	34.45 ± 0.01	−4.71	31.36 ± 0.01	−1.63	−5.34	−2.87	97.13 ± 0.10	−0.69 ± 0.09 [−0.75 ± −0.62]
0.94	LEB2k (p=1)	62.50 ± 0.01	−2.61	60.89 ± 0.00	−0.86	−4.74	−0.77	97.13 ± 0.10	−0.69 ± 0.09 [−0.75 ± −0.62]
0.94	Huffman	33.67 ± 0.01	−3.79	31.28 ± 0.01	−1.28	−7.15	−3.90	97.13 ± 0.10	−0.68 ± 0.08 [−0.74 ± −0.62]

**Table 12:** CAADS on CIFAR-10 with 8-bit delta updates over 50 communication rounds. Values are mean ± sample SD; accuracy differences are paired against static 8-bit quantization with two-sided 95% Student-*t* confidence intervals.

$z_{f}$	Method	Adaptive Up (KiB)	Δ Up (KiB)	Adaptive Down (KiB)	Δ Down (KiB)	$\Delta t_{\mathrm{enc}}$ (ms)	$\Delta t_{\mathrm{dec}}$ (ms)	Final Acc. (%)	Δ Acc. vs. 8b Quant. (pp)
0.80	Elias gamma	1686.07 ± 1.83	+455.34	1328.05 ± 2.30	+173.13	+523.68	+369.58	88.62 ± 0.16	+0.79 ± 0.44 [+0.47 ± +1.10]
0.80	Elias delta	1877.85 ± 1.54	+602.35	1417.62 ± 2.89	+249.81	+688.39	+405.31	88.62 ± 0.16	+0.79 ± 0.44 [+0.47 ± +1.10]
0.80	Golomb–Rice (k=0)	1594.40 ± 3.22	+387.77	1280.73 ± 1.95	+132.60	+597.05	+273.23	88.62 ± 0.16	+0.79 ± 0.44 [+0.47 ± +1.10]
0.80	Exp-Golomb (k=0)	1686.08 ± 1.83	+455.34	1328.06 ± 2.30	+173.13	+348.99	+234.42	88.62 ± 0.16	+0.79 ± 0.44 [+0.47 ± +1.10]
0.80	LEB2k (p=1)	2585.92 ± 2.38	+277.64	2357.19 ± 1.44	+89.33	+556.38	+431.58	88.62 ± 0.16	+0.79 ± 0.44 [+0.47 ± +1.10]
0.80	Huffman	1594.78 ± 3.21	+387.95	1281.05 ± 1.95	+132.73	+693.12	+318.73	88.62 ± 0.16	+0.79 ± 0.44 [+0.47 ± +1.10]
0.94	Elias gamma	1263.38 ± 0.41	+32.64	1164.79 ± 0.64	+9.87	+716.75	+366.79	88.11 ± 0.22	+0.27 ± 0.33 [+0.04 ± +0.50]
0.94	Elias delta	1321.34 ± 0.68	+45.84	1182.44 ± 0.92	+14.63	+561.85	+283.44	88.11 ± 0.22	+0.27 ± 0.33 [+0.04 ± +0.50]
0.94	Golomb–Rice (k=0)	1232.14 ± 0.40	+25.32	1155.57 ± 0.62	+7.38	+62.06	+61.87	87.96 ± 0.19	+0.19 ± 0.26 [−0.14 ± +0.51]
0.94	Exp-Golomb (k=0)	1263.33 ± 0.37	+32.36	1164.90 ± 0.84	+9.90	+61.79	+59.30	87.96 ± 0.19	+0.19 ± 0.26 [−0.14 ± +0.51]
0.94	LEB2k (p=1)	2326.05 ± 0.39	+17.65	2272.71 ± 0.40	+4.80	+129.33	+112.65	87.96 ± 0.19	+0.19 ± 0.26 [−0.14 ± +0.51]
0.94	Huffman	1232.36 ± 0.40	+25.35	1155.78 ± 0.62	+7.41	+95.22	+63.61	87.96 ± 0.19	+0.19 ± 0.26 [−0.14 ± +0.51]
0.96	Elias gamma	1208.04 ± 0.12	−22.69	1147.56 ± 0.31	−7.36	+110.09	+104.24	87.63 ± 0.34	−0.21 ± 0.41 [−0.51 ± +0.08]
0.96	Elias delta	1243.96 ± 0.25	−31.54	1156.89 ± 0.46	−10.92	−164.12	−141.56	87.63 ± 0.34	−0.21 ± 0.41 [−0.51 ± +0.08]
0.96	Golomb–Rice (k=0)	1188.82 ± 0.10	−17.82	1142.63 ± 0.23	−5.50	+70.08	+95.31	87.63 ± 0.34	−0.21 ± 0.41 [−0.51 ± +0.08]
0.96	Exp-Golomb (k=0)	1208.04 ± 0.12	−22.70	1147.53 ± 0.24	−7.39	−178.96	−63.91	87.66 ± 0.30	−0.18 ± 0.38 [−0.45 ± +0.09]
0.96	LEB2k (p=1)	2295.76 ± 0.08	−12.52	2264.25 ± 0.11	−3.61	+190.96	+189.53	87.66 ± 0.30	−0.18 ± 0.38 [−0.45 ± +0.09]
0.96	Huffman	1189.00 ± 0.11	−17.83	1142.80 ± 0.23	−5.51	−359.14	−225.23	87.63 ± 0.34	−0.21 ± 0.41 [−0.51 ± +0.08]

**Table 13:** Nested component ablation of CAADS on MNIST with 8-bit Huffman-coded delta updates. Values are reported as mean ± sample SD.

$z_{f}$	Variant	Uplink (KiB)	Downlink (KiB)	Encode (ms)	Decode (ms)	Accuracy (%)
–	Static (s=0.005)	50.36 ± 0.05	37.68 ± 0.02	117.59 ± 1.28	57.90 ± 0.79	97.94 ± 0.06
0.80	Zero fraction	41.90 ± 0.01	34.20 ± 0.01	107.51 ± 3.11	50.77 ± 0.98	97.91 ± 0.06
0.80	+ drift	44.36 ± 0.01	35.06 ± 0.01	112.51 ± 8.30	53.67 ± 3.37	97.95 ± 0.05
0.80	+ coder-aware	42.11 ± 0.03	34.01 ± 0.02	107.44 ± 5.73	50.76 ± 0.71	97.87 ± 0.06
0.94	Zero fraction	34.79 ± 0.00	31.94 ± 0.00	93.62 ± 2.32	43.57 ± 0.49	97.22 ± 0.05
0.94	+ drift	37.56 ± 0.01	32.66 ± 0.01	99.56 ± 3.36	47.08 ± 1.57	97.81 ± 0.06
0.94	+ coder-aware	33.68 ± 0.01	31.29 ± 0.01	93.09 ± 5.34	42.30 ± 0.47	97.14 ± 0.09

**Table 14:** FEMNIST compression results on native LEAF user partitions over 100 communication rounds. Static 8-bit delta updates use s=0.05. Values are mean ± sample SD across 10 seeds; paired final-accuracy differences are relative to the same-seed raw baseline with two-sided 95% Student-*t* confidence intervals.

Method	Mean Up (KiB)	Up Red. (%)	Down Obj. (KiB)	Down Red. (%)	Enc. (ms)	Dec. (ms)	Final Acc. (%)	Δ Acc. (pp)
Raw reference								
Raw baseline	1676.02 ± 0.00	0.00 ± 0.00	1676.02 ± 0.00	0.00 ± 0.00	–	–	76.77 ± 1.33	+0.00 ± 0.00 [+0.00 ± +0.00]
Static and adaptive delta updates								
8b Quant.	421.22 ± 0.00	74.87 ± 0.00	421.22 ± 0.00	74.87 ± 0.00	30.63 ± 1.99	0.69 ± 0.04	75.99 ± 0.98	−0.78 ± 1.58 [−1.91 ± +0.35]
Elias gamma (static)	59.23 ± 0.59	96.47 ± 0.04	55.08 ± 0.08	96.71 ± 0.00	231.68 ± 1.85	126.77 ± 0.76	75.99 ± 0.98	−0.78 ± 1.58 [−1.91 ± +0.35]
Elias gamma ($z_{f}=0.94$)	54.32 ± 0.02	96.76 ± 0.00	53.82 ± 0.03	96.79 ± 0.00	224.36 ± 0.46	122.71 ± 0.79	75.09 ± 1.31	−1.68 ± 1.64 [−2.85 ± −0.51]
Elias delta (static)	61.76 ± 0.87	96.32 ± 0.05	55.84 ± 0.11	96.67 ± 0.01	270.65 ± 1.50	182.72 ± 2.41	75.99 ± 0.98	−0.78 ± 1.58 [−1.91 ± +0.35]
Elias delta ($z_{f}=0.94$)	54.95 ± 0.02	96.72 ± 0.00	54.22 ± 0.04	96.77 ± 0.00	261.52 ± 0.75	177.04 ± 1.30	75.09 ± 1.31	−1.68 ± 1.64 [−2.85 ± −0.51]
Golomb–Rice (k=0) (static)	57.73 ± 0.45	96.56 ± 0.03	54.32 ± 0.04	96.76 ± 0.00	236.28 ± 1.67	99.23 ± 0.94	75.99 ± 0.98	−0.78 ± 1.58 [−1.91 ± +0.35]
Golomb–Rice (k=0) ($z_{f}=0.94$)	53.83 ± 0.01	96.79 ± 0.00	53.44 ± 0.01	96.81 ± 0.00	226.52 ± 1.13	96.12 ± 1.22	75.09 ± 1.31	−1.68 ± 1.64 [−2.85 ± −0.51]
Exp-Golomb (k=0) (static)	59.24 ± 0.59	96.47 ± 0.04	55.08 ± 0.08	96.71 ± 0.00	236.99 ± 1.11	142.85 ± 1.02	75.99 ± 0.98	−0.78 ± 1.58 [−1.91 ± +0.35]
Exp-Golomb (k=0) ($z_{f}=0.94$)	54.33 ± 0.02	96.76 ± 0.00	53.83 ± 0.03	96.79 ± 0.00	230.09 ± 1.13	139.32 ± 1.18	75.09 ± 1.31	−1.68 ± 1.64 [−2.85 ± −0.51]
LEB2k (p=1) (static)	108.49 ± 0.32	93.53 ± 0.02	105.89 ± 0.02	93.68 ± 0.00	289.65 ± 1.34	193.13 ± 0.85	75.99 ± 0.98	−0.78 ± 1.58 [−1.91 ± +0.35]
LEB2k (p=1) ($z_{f}=0.94$)	105.67 ± 0.02	93.70 ± 0.00	105.39 ± 0.00	93.71 ± 0.00	277.58 ± 0.79	191.18 ± 2.86	75.09 ± 1.31	−1.68 ± 1.64 [−2.85 ± −0.51]
Huffman (static)	57.83 ± 0.44	96.55 ± 0.03	54.37 ± 0.05	96.76 ± 0.00	187.21 ± 1.60	74.60 ± 0.77	75.99 ± 0.98	−0.78 ± 1.58 [−1.91 ± +0.35]
Huffman ($z_{f}=0.94$)	53.90 ± 0.01	96.78 ± 0.00	53.49 ± 0.01	96.81 ± 0.00	177.90 ± 0.43	73.30 ± 0.57	75.09 ± 1.31	−1.68 ± 1.64 [−2.85 ± −0.51]
Native competitive baselines								
FedPAQ	261.91 ± 0.00	84.37 ± 0.00	1675.74 ± 0.00	0.02 ± 0.00	202.71 ± 22.88	306.31 ± 34.61	57.40 ± 4.91	−19.37 ± 4.70 [−22.73 ± −16.01]
FedBAT	53.96 ± 0.00	96.78 ± 0.00	1675.74 ± 0.00	0.02 ± 0.00	76.88 ± 9.32	74.40 ± 5.44	54.48 ± 4.24	−22.29 ± 4.57 [−25.55 ± −19.02]
SparsyFed	129.09 ± 2.66	92.30 ± 0.16	169.91 ± 0.00	89.86 ± 0.00	1.01 ± 0.18	0.42 ± 0.03	5.50 ± 0.24	−71.27 ± 1.34 [−72.23 ± −70.31]

**Table 15:** Sent140 compression results on native LEAF user partitions over 100 communication rounds. Static 8-bit delta updates use s=0.0001. Values are mean ± sample SD across 10 seeds; paired final-accuracy differences are relative to the same-seed raw baseline with two-sided 95% Student-*t* confidence intervals.

Method	Mean Up (KiB)	Up Red. (%)	Down Obj. (KiB)	Down Red. (%)	Enc. (ms)	Dec. (ms)	Final Acc. (%)	Δ Acc. (pp)
Raw reference								
Raw baseline	79.13 ± 0.00	0.00 ± 0.00	79.13 ± 0.00	0.00 ± 0.00	–	–	57.54 ± 4.06	+0.00 ± 0.00 [+0.00 ± +0.00]
Static and adaptive delta updates								
8b Quant.	–	–	20.57 ± 0.00	74.01 ± 0.00	–	–	58.76 ± 1.01	+1.22 ± 3.43 [−1.23 ± +3.67]
Elias gamma (static)	2.89 ± 0.00	96.34 ± 0.00	2.89 ± 0.00	96.35 ± 0.00	9.84 ± 0.13	5.53 ± 0.04	58.76 ± 1.01	+1.22 ± 3.43 [−1.23 ± +3.67]
Elias gamma ($z_{f}=0.94$)	2.90 ± 0.00	96.34 ± 0.00	2.89 ± 0.00	96.35 ± 0.00	9.76 ± 0.08	5.36 ± 0.07	56.93 ± 3.33	−0.61 ± 0.88 [−1.24 ± +0.02]
Elias delta (static)	2.89 ± 0.00	96.34 ± 0.00	2.89 ± 0.00	96.35 ± 0.00	11.38 ± 0.09	8.05 ± 0.08	58.76 ± 1.01	+1.22 ± 3.43 [−1.23 ± +3.67]
Elias delta ($z_{f}=0.94$)	2.90 ± 0.00	96.34 ± 0.00	2.89 ± 0.00	96.35 ± 0.00	11.48 ± 0.08	7.95 ± 0.14	56.93 ± 3.33	−0.61 ± 0.88 [−1.24 ± +0.02]
Golomb–Rice (k=0) (static)	2.90 ± 0.00	96.34 ± 0.00	2.90 ± 0.00	96.34 ± 0.00	9.71 ± 0.08	4.26 ± 0.05	58.76 ± 1.01	+1.22 ± 3.43 [−1.23 ± +3.67]
Golomb–Rice (k=0) ($z_{f}=0.94$)	2.90 ± 0.00	96.33 ± 0.00	2.90 ± 0.00	96.34 ± 0.00	9.83 ± 0.08	4.19 ± 0.07	56.93 ± 3.33	−0.61 ± 0.88 [−1.24 ± +0.02]
Exp-Golomb (k=0) (static)	2.90 ± 0.00	96.34 ± 0.00	2.89 ± 0.00	96.34 ± 0.00	9.82 ± 0.13	6.32 ± 0.06	58.76 ± 1.01	+1.22 ± 3.43 [−1.23 ± +3.67]
Exp-Golomb (k=0) ($z_{f}=0.94$)	2.90 ± 0.00	96.33 ± 0.00	2.89 ± 0.00	96.34 ± 0.00	10.01 ± 0.11	6.18 ± 0.08	56.93 ± 3.33	−0.61 ± 0.88 [−1.24 ± +0.02]
LEB2k (p=1) (static)	5.35 ± 0.00	93.24 ± 0.00	5.35 ± 0.00	93.24 ± 0.00	12.53 ± 0.07	8.68 ± 0.10	58.76 ± 1.01	+1.22 ± 3.43 [−1.23 ± +3.67]
LEB2k (p=1) ($z_{f}=0.94$)	5.35 ± 0.00	93.23 ± 0.00	5.35 ± 0.00	93.24 ± 0.00	12.57 ± 0.11	8.57 ± 0.08	56.93 ± 3.33	−0.61 ± 0.88 [−1.24 ± +0.02]
Huffman (static)	2.95 ± 0.00	96.27 ± 0.00	2.93 ± 0.00	96.29 ± 0.00	7.55 ± 0.04	3.36 ± 0.03	58.76 ± 1.01	+1.22 ± 3.43 [−1.23 ± +3.67]
Huffman ($z_{f}=0.94$)	2.96 ± 0.00	96.26 ± 0.00	2.94 ± 0.00	96.29 ± 0.00	7.77 ± 0.04	3.28 ± 0.05	56.93 ± 3.33	−0.61 ± 0.88 [−1.24 ± +0.02]
Native competitive baselines								
FedPAQ	12.38 ± 0.00	84.36 ± 0.00	78.84 ± 0.00	0.36 ± 0.00	8.07 ± 0.23	12.12 ± 0.32	57.62 ± 3.96	+0.08 ± 0.23 [−0.08 ± +0.25]
FedBAT	2.66 ± 0.00	96.64 ± 0.00	78.84 ± 0.00	0.36 ± 0.00	3.08 ± 0.05	3.37 ± 0.06	53.64 ± 1.59	−3.89 ± 5.13 [−7.57 ± −0.22]
SparsyFed	0.80 ± 0.03	98.99 ± 0.03	8.00 ± 0.00	89.89 ± 0.00	0.17 ± 0.01	0.04 ± 0.00	50.86 ± 2.68	−6.68 ± 4.31 [−9.76 ± −3.60]

Uplink is mean payload per client; downlink is one encoded server object. Accuracy differences are paired against the same-seed raw baseline. Enc./Dec. are absolute mean per-client uplink codec times. FedPAQ and FedBAT retain dense full-precision downlinks; SparsyFed transmits a sparse global model. Sent140 quantization uplink size and separate encode/decode times were not recorded and are shown as –.

**Table 16:** Mean total codec time and derived break-even bandwidth for the server measurements and Raspberry Pi replay measurements. Server values use the measurements reported in the preceding subsections; Raspberry Pi values use the 10-repeat replay experiments. Break-even bandwidth is computed for one transmitted update object.

Dataset	Platform	Method	$T_{\mathrm{codec}}$ (ms)	$R_{\mathrm{break}}$ (Mbit/s)
MNIST	Server	8b Quant.	19.13	305.59
MNIST	Server	Huffman	160.49	11.89
MNIST	Server	Huffman ($z_{f}=0.80$)	177.38	10.38
MNIST	Server	Huffman ($z_{f}=0.94$)	149.44	13.14
MNIST	Pi 5	8b Quant.	34.05	170.66
MNIST	Pi 5	Huffman	313.85	6.20
MNIST	Pi 5	Huffman ($z_{f}=0.80$)	323.01	6.29
MNIST	Pi 5	Huffman ($z_{f}=0.94$)	327.70	6.19
MNIST	Pi 3B	8b Quant.	249.45	23.29
MNIST	Pi 3B	Huffman	2377.27	0.82
MNIST	Pi 3B	Huffman ($z_{f}=0.80$)	2472.59	0.82
MNIST	Pi 3B	Huffman ($z_{f}=0.94$)	2468.23	0.82
FEMNIST	Server	8b Quant.	31.32	328.20
FEMNIST	Server	Huffman	261.81	12.92
FEMNIST	Server	Huffman ($z_{f}=0.94$)	251.20	13.69
FEMNIST	Pi 5	8b Quant.	81.02	126.61
FEMNIST	Pi 5	Huffman	668.84	4.93
FEMNIST	Pi 5	Huffman ($z_{f}=0.94$)	752.56	5.50
FEMNIST	Pi 3B	8b Quant.	548.67	18.69
FEMNIST	Pi 3B	Huffman	4936.68	0.66
FEMNIST	Pi 3B	Huffman ($z_{f}=0.94$)	5765.66	0.72
CIFAR-10	Server	8b Quant.	678.81	326.27
CIFAR-10	Server	Huffman	5223.68	14.08
CIFAR-10	Server	Huffman ($z_{f}=0.80$)	6235.53	10.95
CIFAR-10	Server	Huffman ($z_{f}=0.94$)	5382.51	13.56
CIFAR-10	Server	Huffman ($z_{f}=0.96$)	4639.31	16.20
CIFAR-10	Pi 5	8b Quant.	1141.51	193.96
CIFAR-10	Pi 5	Huffman	11,873.81	6.02
CIFAR-10	Pi 5	Huffman ($z_{f}=0.80$)	12,186.38	6.09
CIFAR-10	Pi 5	Huffman ($z_{f}=0.94$)	12,722.01	5.98
CIFAR-10	Pi 5	Huffman ($z_{f}=0.96$)	13,796.82	5.97

**Table 17:** Communication-plus-codec time savings at representative bandwidths. Positive values indicate lower total time than the corresponding reference configuration.

Dataset	Platform	Method	S(1) (s)	S(5) (s)	S(15) (s)	S(25) (s)	S(100) (s)
MNIST	Server	8b Quant.	+5.831	+1.151	+0.371	+0.215	+0.039
MNIST	Server	Static Huffman	+1.546	+0.196	−0.029	−0.074	−0.125
MNIST	Server	Coder-aware ($z_{f}=0.94$)	+1.594	+0.214	−0.016	−0.062	−0.113
MNIST	Pi 5	8b Quant.	+5.778	+1.128	+0.353	+0.198	+0.024
MNIST	Pi 5	Static Huffman	+1.446	+0.066	−0.164	−0.210	−0.262
MNIST	Pi 5	Coder-aware ($z_{f}=0.94$)	+1.445	+0.065	−0.165	−0.211	−0.263
MNIST	Pi 3B	8b Quant.	+5.563	+0.913	+0.138	−0.017	−0.191
MNIST	Pi 3B	Static Huffman	−0.402	−1.782	−2.012	−2.058	−2.110
MNIST	Pi 3B	Coder-aware ($z_{f}=0.94$)	−0.395	−1.775	−2.005	−2.051	−2.103
FEMNIST	Server	8b Quant.	+10.244	+2.024	+0.654	+0.380	+0.071
FEMNIST	Server	Static Huffman	+2.733	+0.363	−0.032	−0.111	−0.201
FEMNIST	Server	Coder-aware ($z_{f}=0.94$)	+2.816	+0.386	−0.019	−0.100	−0.190
FEMNIST	Pi 5	8b Quant.	+10.193	+1.973	+0.603	+0.329	+0.022
FEMNIST	Pi 5	Static Huffman	+2.336	−0.004	−0.394	−0.472	−0.559
FEMNIST	Pi 5	Coder-aware ($z_{f}=0.94$)	+2.456	+0.056	−0.344	−0.424	−0.513
FEMNIST	Pi 3B	8b Quant.	+9.690	+1.500	+0.135	−0.138	−0.446
FEMNIST	Pi 3B	Static Huffman	−1.500	−3.810	−4.195	−4.272	−4.359
FEMNIST	Pi 3B	Coder-aware ($z_{f}=0.94$)	−1.125	−3.525	−3.925	−4.005	−4.095
CIFAR-10	Server	8b Quant.	+220.796	+43.616	+14.086	+8.180	+1.536
CIFAR-10	Server	Static Huffman	+59.468	+8.258	−0.277	−1.984	−3.905
CIFAR-10	Server	Coder-aware ($z_{f}=0.94$)	+59.120	+8.060	−0.450	−2.152	−4.066
CIFAR-10	Pi 5	8b Quant.	+220.259	+43.139	+13.619	+7.715	+1.073
CIFAR-10	Pi 5	Static Huffman	+53.917	+2.197	−6.423	−8.147	−10.086
CIFAR-10	Pi 5	Coder-aware ($z_{f}=0.94$)	+53.691	+2.121	−6.474	−8.193	−10.127

## Data Availability

The FEDCODE implementation, experiment scripts, and run configurations used to reproduce the results in this paper are available at: https://github.com/Guber/fed-code (accessed on 27 September 2026).

## Abbreviations

FL	Federated Learning
IoT	Internet of Things
CNN	Convolutional Neural Network
LSTM	Long Short-Term Memory
LEAF	Benchmark framework for learning in federated settings

## Appendix A. Convergence Analysis of the CAADS Adaptive Quantizer

This appendix analyzes how quantization error from the CAADS adaptive delta-scale quantizer propagates through repeated FedAvg aggregation under Non-IID data. The analysis uses standard smoothness and stochastic-gradient assumptions for heterogeneous FedAvg [88], while the quantization-error bound is specific to CAADS. We consider equal-weight FedAvg over *K* participating clients, make no unbiasedness assumption on the quantizer, and carry its bounded error directly through the descent argument. Expectations are taken over the stochastic local gradients conditional on the global iterate $w^{t}$. Importantly, the analysis does not require a separate uniform upper bound on the stochastic-gradient second moment. Instead, the local-gradient second-moment terms are retained explicitly in the derivation and controlled through the sufficient conditions on the quantization error and local learning rate derived below.

### Appendix A.1. Assumptions

**Assumption A1** (No quantizer saturation)**.**
*For the selected scale $s^{★}$, the update values lie within the representable range of the quantizer:*

(A1)
$$\max_{i}|{\left(\Delta w\right)}_{i}|\leq s^{★}\max\{|q_{\min}|,q_{\max}\}.$$

**Assumption A2** (*L*-smooth objectives)**.**

*The global objective F(w) and each local objective $F_{k}\left(w\right)$ are differentiable and L-smooth. In particular, for any x,y,*

(A2)
$$F\left(y\right)\leq F\left(x\right)+\langle \nabla F\left(x\right),y-x\rangle +\frac{L}{2}{∥y-x∥}^{2},$$

*and the analogous inequality holds for each $F_{k}$.*

**Assumption A3** (Unbiased stochastic gradients with bounded variance)**.**
*For every client k,*

(A3) $$E\left[g_{k}\left(w\right)\right]=\nabla F_{k}\left(w\right),\;E\left[∥g_{k}\left(w\right)-\nabla F_{k}{\left(w\right)∥}^{2}\right]\leq \sigma^{2}.$$

*No separate uniform bound on $E∥g_{k}{\left(w\right)∥}^{2}$ is assumed; the local-gradient second-moment terms are retained explicitly and controlled under the sufficient conditions in *(A34) *and* (A35)*.*

**Assumption A4** (Bounded Non-IID heterogeneity)**.**

*The client objectives satisfy*

(A4)
$$\frac{1}{K}\sum_{k=1}^{K}{∥\nabla F_{k}\left(w\right)-\nabla F\left(w\right)∥}^{2}\leq \kappa^{2}.$$

### Appendix A.2. Quantization Error Bound

**Lemma** **A1.**
*Under Assumption A1 and the conditions of Algorithm 1, there exist constants Ψ,δ>0 such that*

(A5)
$${∥Q\left(\Delta w\right)-\Delta w∥}^{2}\leq \Psi +\delta {∥\Delta w∥}^{2}.$$

**Proof.** Algorithm 1 maps $\Delta w\in R^{N}$ to the dequantized vector $Q(\Delta w,s^{★})$. Under Assumption A1, rounding introduces at most half a quantization step per coordinate:

(A6) $$|Q\left({\left(\Delta w\right)}_{i},s^{★}\right)-{\left(\Delta w\right)}_{i}|\leq \frac{s^{★}}{2}.$$

Therefore,

(A7) $$∥Q\left(\Delta w,s^{★}\right){-\Delta w∥}^{2}\leq \frac{N}{4}{\left(s^{★}\right)}^{2}.$$

Let $m_{\max}=\max\left(M\right)$. By definition, $s^{★}\leq s_{\max}$. As in the supplied scale-selection argument, assume that at least one candidate multiplier m∈M satisfies $ms_{\mathrm{prop}}\geq s_{\min}$. Then, $s^{★}\leq m_{\max}s_{\mathrm{prop}}$. From Algorithm 1 and the clipping operation,

(A8) $$\begin{array}{cc} s_{\mathrm{prop}}\; & \leq \left(1-\lambda \right)\left[\left(1-\eta \right)s_{\mathrm{prev}}+\eta s_{\mathrm{prev}}\exp\left(\rho_{\mathrm{target}}-\rho_{0}\left(s_{\mathrm{prev}}\right)\right)\right]+\lambda \alpha d. \end{array}$$

Because $\rho_{0}\left(s_{\mathrm{prev}}\right)\geq 0$ and $s_{\mathrm{prev}}\leq s_{\max}$,

(A9) $$s_{\mathrm{prop}}\leq C+\lambda \alpha d,\;C=\left(1-\lambda \right)s_{\max}\left[\left(1-\eta \right)+\eta \exp\left(\rho_{\mathrm{target}}\right)\right].$$

Using the RMS definition from Algorithm 1, $d=∥\Delta w∥/\sqrt{N}$, hence,

(A10) $$s_{\mathrm{prop}}\leq C+\frac{\lambda \alpha }{\sqrt{N}}∥\Delta w∥.$$

Combining (A7)–(A10) gives

(A11) $$∥Q\left(\Delta w,s^{★}\right){-\Delta w∥}^{2}\leq \frac{N}{4}m_{\max}^{2}{\left(C+\frac{\lambda \alpha }{\sqrt{N}}∥\Delta w∥\right)}^{2}.$$

Since ${\left(a+b\right)}^{2}\leq 2a^{2}+2b^{2}$,

(A12) $$∥Q\left(\Delta w,s^{★}\right){-\Delta w∥}^{2}\leq \frac{Nm_{\max}^{2}C^{2}}{2}+\frac{m_{\max}^{2}\lambda^{2}\alpha^{2}}{2}{∥\Delta w∥}^{2}.$$

Together with $s^{★}\leq s_{\max}$, this yields

(A13) $$∥Q\left(\Delta w,s^{★}\right){-\Delta w∥}^{2}\leq \min\left\{\frac{N}{4}s_{\max}^{2},\Psi +\delta {∥\Delta w∥}^{2}\right\},$$

where

(A14) $$\delta =\frac{m_{\max}^{2}\lambda^{2}\alpha^{2}}{2},\;\Psi =\frac{Nm_{\max}^{2}}{2}{\left(\left(1-\lambda \right)s_{\max}\left[\left(1-\eta \right)+\eta \exp\left(\rho_{\mathrm{target}}\right)\right]\right)}^{2}.$$

□

### Appendix A.3. FedAvg Update Rule

Let $w^{t}$ denote the global model parameters in round *t*. Client *k* performs *E* local steps with learning rate $\eta_{l}$. Its uncompressed update is

(A15) $$\Delta w_{k}^{t}=w_{k}^{t,E}-w^{t}=-\eta_{l}\sum_{e=1}^{E}g_{k}^{t,e}.$$

The adaptive quantizer produces ${\hat{\Delta w}}_{k}^{t}=Q\left(\Delta w_{k}^{t},s_{k}^{★}\right)$, and the server update is

(A16) $$w^{t+1}=w^{t}+\frac{1}{K}\sum_{k=1}^{K}{\hat{\Delta w}}_{k}^{t}.$$

Define

(A17) $$\Delta w^{t}=\frac{1}{K}\sum_{k=1}^{K}\Delta w_{k}^{t},\;e^{t}=\frac{1}{K}\sum_{k=1}^{K}\left(Q\left(\Delta w_{k}^{t},s_{k}^{★}\right)-\Delta w_{k}^{t}\right).$$

Then,

(A18) $$w^{t+1}=w^{t}+\Delta w^{t}+e^{t}.$$

The term $e^{t}$ is retained explicitly; no zero-mean or unbiasedness assumption is made for the quantization error.

### Appendix A.4. Descent Expansion

Applying *L*-smoothness to (A18),

(A19) $$F\left(w^{t+1}\right)\leq F\left(w^{t}\right)+\langle \nabla F\left(w^{t}\right),\Delta w^{t}+e^{t}\rangle +\frac{L}{2}{∥\Delta w^{t}+e^{t}∥}^{2}.$$

Taking conditional expectation and using Young’s inequality with any β>0,

(A20) $$E\langle \nabla F\left(w^{t}\right),e^{t}\rangle \leq \frac{\beta }{2}∥\nabla F\left(w^{t}\right){∥}^{2}+\frac{1}{2\beta }E{∥e^{t}∥}^{2},$$

and

(A21) $$\frac{L}{2}E∥\Delta w^{t}+e^{t}{∥}^{2}\leq LE∥\Delta w^{t}{∥}^{2}+LE{∥e^{t}∥}^{2}.$$

Consequently,

(A22) $$\begin{array}{cc} E[F\left(w^{t+1}\right)]\leq & F\left(w^{t}\right)+E\langle \nabla F\left(w^{t}\right),\Delta w^{t}\rangle +\frac{\beta }{2}{∥\nabla F\left(w^{t}\right)∥}^{2}\\ & +\left(\frac{1}{2\beta }+L\right)E∥e^{t}{∥}^{2}+LE{∥\Delta w^{t}∥}^{2}. \end{array}$$

By Jensen’s inequality and Lemma A1,

(A23) $$E∥e^{t}{∥}^{2}\leq \Psi +\frac{\delta }{K}\sum_{k=1}^{K}E{∥\Delta w_{k}^{t}∥}^{2}.$$

For the local updates, Jensen’s inequality and Assumption A3 give

(A24) $$\begin{array}{cc} E∥\Delta w_{k}^{t}{∥}^{2} & =E{∥-\eta_{l}\sum_{e=1}^{E}g_{k}^{t,e}∥}^{2}\\ & \leq \eta_{l}^{2}E\sum_{e=1}^{E}E{∥g_{k}^{t,e}∥}^{2}\\ & \leq \eta_{l}^{2}E\left(E\sigma^{2}+\sum_{e=1}^{E}E{∥\nabla F_{k}\left(w_{k}^{t,e}\right)∥}^{2}\right). \end{array}$$

Thus,

(A25) $$E∥e^{t}{∥}^{2}\leq \Psi +\delta \eta_{l}^{2}E\left(E\sigma^{2}+\frac{1}{K}\sum_{k=1}^{K}\sum_{e=1}^{E}E{∥\nabla F_{k}\left(w_{k}^{t,e}\right)∥}^{2}\right),$$

and similarly

(A26) $$LE∥\Delta w^{t}{∥}^{2}\leq L\eta_{l}^{2}E\left(E\sigma^{2}+\frac{1}{K}\sum_{k=1}^{K}\sum_{e=1}^{E}E{∥\nabla F_{k}\left(w_{k}^{t,e}\right)∥}^{2}\right).$$

For the descent term, using $-\langle a,b\rangle =-\frac{1}{2}{(∥a∥}^{2}+{∥b∥}^{2})+\frac{1}{2}{∥a-b∥}^{2}$,

(A27) $$\begin{array}{cc} E\langle \nabla F\left(w^{t}\right),\Delta w^{t}\rangle = & -\frac{\eta_{l}E}{2}∥\nabla F\left(w^{t}\right){∥}^{2}-\frac{\eta_{l}}{2K}\sum_{k,e}E{∥\nabla F_{k}\left(w_{k}^{t,e}\right)∥}^{2}\\ & +\frac{\eta_{l}}{2K}\sum_{k,e}E{∥\nabla F\left(w^{t}\right)-\nabla F_{k}\left(w_{k}^{t,e}\right)∥}^{2}. \end{array}$$

For each k,e,

(A28) $$\begin{array}{cc} ∥\nabla F\left(w^{t}\right)-\nabla F_{k}\left(w_{k}^{t,e}\right){∥}^{2}\leq & 2{∥\nabla F\left(w^{t}\right)-\nabla F_{k}\left(w^{t}\right)∥}^{2}\\ & +2∥\nabla F_{k}\left(w^{t}\right)-\nabla F_{k}\left(w_{k}^{t,e}\right){∥}^{2}. \end{array}$$

After summing over clients, the first term is controlled by Assumption A4. By *L*-smoothness of $F_{k}$,

(A29) $$∥\nabla F_{k}\left(w^{t}\right)-\nabla F_{k}\left(w_{k}^{t,e}\right){∥}^{2}\leq L^{2}{∥w^{t}-w_{k}^{t,e}∥}^{2}.$$

Since $w_{k}^{t,e}$ is obtained after at most *E* local steps from $w^{t}$,

(A30) $$E∥w^{t}-w_{k}^{t,e}{∥}^{2}\leq \eta_{l}^{2}E\left(E\sigma^{2}+\sum_{i=1}^{E}E{∥\nabla F_{k}\left(w_{k}^{t,i}\right)∥}^{2}\right).$$

Substituting these bounds into (A27) yields

(A31) $$\begin{array}{cc} E\langle \nabla F\left(w^{t}\right),\Delta w^{t}\rangle \leq & -\frac{\eta_{l}E}{2}{∥\nabla F\left(w^{t}\right)∥}^{2}+\eta_{l}E\kappa^{2}+\eta_{l}^{3}E^{3}L^{2}\sigma^{2}\\ & -\frac{\eta_{l}}{K}\left(\frac{1}{2}-\eta_{l}^{2}L^{2}E^{2}\right)\sum_{k,e}E{∥\nabla F_{k}\left(w_{k}^{t,e}\right)∥}^{2}. \end{array}$$

### Appendix A.5. Bound over Multiple Communication Rounds

Applying (A25), (A26), and (A31) to (A22) gives

(A32) $$\begin{array}{cc} E[F\left(w^{t+1}\right)]\leq & F\left(w^{t}\right)-\frac{\eta_{l}E}{2}{∥\nabla F\left(w^{t}\right)∥}^{2}\\ & +\left(\Psi +\delta \eta_{l}^{2}E^{2}\sigma^{2}\right)\left(\frac{1}{2\beta }+L\right)+L\eta_{l}^{2}E^{2}\sigma^{2}+\eta_{l}E\kappa^{2}+L^{2}\eta_{l}^{3}E^{3}\sigma^{2}\\ & +\left[\left(\frac{1}{2\beta }+L\right)\delta \eta_{l}^{2}E+L\eta_{l}^{2}E-\eta_{l}\left(\frac{1}{2}-L^{2}\eta_{l}^{2}E^{2}\right)\right]\frac{1}{K}\sum_{k,e}E{∥\nabla F_{k}\left(w_{k}^{t,e}\right)∥}^{2}. \end{array}$$

Choose

(A33) $$\beta =\frac{\eta_{l}E}{2}.$$

Then, the coefficient of ${∥\nabla F\left(w^{t}\right)∥}^{2}$ becomes $-\eta_{l}E/4$. To make the coefficient of the summed local-gradient second-moment term non-positive, the proof requires

(A34) $$\delta <\frac{1}{2},$$

and, provided that condition holds,

(A35) $$\eta_{l}\leq \frac{-\left(1+\delta \right)+\sqrt{{\left(1+\delta \right)}^{2}+4\left(\frac{1}{2}-\delta \right)}}{2LE}.$$

Thus, the analysis does not require a separate global upper bound on $E∥g_{k}{\left(w\right)∥}^{2}$; instead, the corresponding second-moment contribution is controlled directly through the above sufficient conditions.

Under these conditions,

(A36) $$\begin{array}{cc} \frac{\eta_{l}E}{4}{∥\nabla F\left(w^{t}\right)∥}^{2}\leq & F\left(w^{t}\right)-E\left[F\left(w^{t+1}\right)\right]+\eta_{l}E\kappa^{2}\\ & +\eta_{l}E\sigma^{2}\left[\delta +L\eta_{l}E\left(1+\delta \right)+L^{2}\eta_{l}^{2}E^{2}\right]+\left(\frac{1}{\eta_{l}E}+L\right)\Psi . \end{array}$$

The non-vanishing error floor therefore comprises contributions from data heterogeneity ($\kappa^{2}$), stochastic-gradient variance ($\sigma^{2}$), and bounded adaptive quantization error (Ψ).

Let $F^{★}=\inf_{w}F\left(w\right)$. Taking total expectation in (A36), summing for t=0,…,T−1, and using the telescoping relation

(A37) $$\begin{array}{cc} \sum_{t=0}^{T-1}\left(E\left[F\left(w^{t}\right)\right]-E\left[F\left(w^{t+1}\right)\right]\right)\; & =F\left(w^{0}\right)-E\left[F\left(w^{T}\right)\right]\\ & \leq F\left(w^{0}\right)-F^{★}, \end{array}$$

gives

(A38) $$\begin{array}{cc} \frac{1}{T}\sum_{t=0}^{T-1}E{∥\nabla F\left(w^{t}\right)∥}^{2}\leq & \frac{4\left(F\left(w^{0}\right)-F^{★}\right)}{\eta_{l}ET}+4\kappa^{2}\\ & +4\sigma^{2}\left[\delta +L\eta_{l}E\left(1+\delta \right)+L^{2}\eta_{l}^{2}E^{2}\right]\\ & +\frac{4}{\eta_{l}E}\left(\frac{1}{\eta_{l}E}+L\right)\Psi . \end{array}$$

Under (A34)–(A35), (A38) gives an O(1/T) optimization term and convergence of the average expected squared gradient norm to a bounded neighborhood of zero. The residual depends on client heterogeneity, stochastic-gradient variance, and bounded quantization error. This result applies to the potentially biased adaptive CAADS quantizer through the error bound in Lemma A.1 and does not require an unbiased quantizer or a separate uniform bound on the stochastic-gradient second moment. The quantization-dependent condition is

(A39) $$\begin{array}{c} \delta =\frac{m_{\max}^{2}\lambda^{2}\alpha^{2}}{2}<\frac{1}{2} \end{array}.$$
